# Decreased and Increased Anisotropy along Major Cerebral White Matter Tracts in Preterm Children and Adolescents

**DOI:** 10.1371/journal.pone.0142860

**Published:** 2015-11-11

**Authors:** Katherine E. Travis, Jenna N. Adams, Michal Ben-Shachar, Heidi M. Feldman

**Affiliations:** 1 Division of Neonatal and Developmental Medicine, Department of Pediatrics, Stanford University School of Medicine, Palo Alto, CA, 94303, United States of America; 2 The Gonda Brain Research Center, Bar Ilan University, Ramat Gan, 5290002, Israel; 3 Department of English Literature and Linguistics, Bar Ilan University, Ramat Gan, 5290002, Israel; Beijing Normal University, CHINA

## Abstract

Premature birth is highly prevalent and associated with neurodevelopmental delays and disorders. Adverse outcomes, particularly in children born before 32 weeks of gestation, have been attributed in large part to white matter injuries, often found in periventricular regions using conventional imaging. To date, tractography studies of white matter pathways in children and adolescents born preterm have evaluated only a limited number of tracts simultaneously. The current study compares diffusion properties along 18 major cerebral white matter pathways in children and adolescents born preterm (n = 27) and full term (n = 19), using diffusion magnetic resonance imaging and tractography. We found that compared to the full term group, the preterm group had significantly decreased FA in segments of the bilateral uncinate fasciculus and anterior segments of the right inferior fronto-occipital fasciculus. Additionally, the preterm group had significantly increased FA in segments of the right and left anterior thalamic radiations, posterior segments of the right inferior fronto-occipital fasciculus, and the right and left inferior longitudinal fasciculus. Increased FA in the preterm group was generally associated with decreased radial diffusivity. These findings indicate that prematurity-related white matter differences in later childhood and adolescence do not affect all tracts in the periventricular zone and can involve both decreased and increased FA. Differences in the patterns of radial diffusivity and axial diffusivity suggest that the tissue properties underlying group FA differences may vary within and across white matter tracts. Distinctive diffusion properties may relate to variations in the timing of injury in the neonatal period, extent of white matter dysmaturity and/or compensatory processes in childhood.

## Introduction

Preterm birth is a significant public health challenge, affecting approximately 12% of the infants born in the US [1]. Complications of preterm birth extend far beyond the newborn period because affected children may experience adverse neurodevelopmental outcomes in multiple developmental domains. Those born before 32 weeks gestation, approximately 1.5% of the population of infants, are at substantial risk for adverse neurodevelopmental delays and disorders such as cerebral palsy, compromised intelligence, attention deficit hyperactivity disorder, and weak visual-spatial, language, and executive function skills [2], though even in this subgroup many children seem unaffected. Unfavorable outcomes have been attributed in large part to injury to the white matter of the brain [3–5]. Diffusion magnetic resonance imaging (dMRI) has proven to be a particularly useful technique for interrogating white matter status in children born preterm [6–8]. Several studies have used dMRI with individualized tractography for precise identification and characterization of specific tracts [9–13], but most studies limit the interrogation to a small number of white matter tracts.

In the present study, we used dMRI with tractography to simultaneously evaluate diffusion properties along 18 multiple major cerebral white matter pathways in a sample of children and adolescents who had been born preterm, in comparison to age-matched peers born full term. We used this ‘wide lens’ approach to test two common assumptions about the neural consequences of premature birth. First, we examined whether white matter impairments occur throughout periventricular regions of white matter and more diffusely in tracts non-adjacent to the lateral ventricles. Second, we examined whether prematurity is associated primarily with reductions in white matter anisotropy. A direct test of these common assumptions is important to improve our understanding regarding white matter development in children born preterm as well as full term. In addition, the results have important implications for understanding how white matter pathways contribute to the wide range of functional outcomes in children and adolescents born preterm.

In the past, the most common form of white matter injury involved cystic lesions, usually found in the periventricular zone, and therefore called periventricular leukomalacia (PVL). In the current era of improved neonatal medical care, the prevalence of cystic lesions has dramatically declined [14] but non-cystic lesions have been found in similar locations and called periventricular white matter injuries (PWMI) [4]. Such white matter injury has been attributed to the vulnerability of the oligodendrocyte precursor line, the cells that are ultimately responsible for myelination. Between 23 and 32 weeks gestation, oligodendrocyte progenitor cells are highly vulnerable to common complications of preterm birth, including hypoxia-ischemia and systemic infection or inflammation [15, 16]. In the aftermath of such injuries, regenerated oligodendrocyte progenitors also show arrested maturation, with failure to fully differentiate and to myelinate axons [16, 17].

The predilection for injury in periventricular regions is thought to arise from the high proportion of oligodendrocyte progenitor cells in these areas [15]. In animal models of prematurity, regional variation in the degree of oligodendrocyte maturity has been observed to coincide with the severity and distribution of hypomyelination [18]. Such findings suggest that the distribution of white matter injuries may also include non-periventricular regions and may be related to the timing of the acute insult or the nature and duration of chronic processes [16, 17]. However, the extent of diffuse white matter injuries in non-periventricular white matter regions has yet to be fully characterized.

Diffusion MRI has become the most common method for detecting and quantifying white matter microstructural changes associated with clinical conditions, such as preterm birth. White matter microstructure is typically quantified by calculating fractional anisotropy (FA), a scalar value that indexes the degree of directional preference in water diffusion [19, 20]. FA can be decomposed into axial diffusivity (AD) and radial diffusivity (RD), which quantify the speed of water diffusion along the principal and perpendicular diffusion directions, respectively. Mean diffusivity (MD) is a measure for the average rate of water displacement within a voxel. Within white matter, increased levels of FA have generally been associated with favorable neurobiological factors, such as increased myelination, greater axonal count and axonal density [21, 22]. It has been argued that RD reflects myelin content and AD reflects axonal status [23–27], but such interpretations for diffusion properties may be oversimplified [22, 28]. Other tissue factors may have counteracting effects on FA, such as directional coherence and the proportion of crossing-fibers within a voxel [29].

Some of the first dMRI studies to evaluate white matter status in children born preterm involved voxel-based analyses. At term equivalent age, these studies have generally found decreased FA and/or increased RD in preterm compared to the full term born neonates [6], consistent with reduced myelin content in the preterm samples. However, increased FA in specific regions has also been reported [30, 31]. Differences in diffusion properties persist from the neonatal period into childhood [32–34], adolescence [35–38] and adulthood [39, 40]. Studies of younger children have generally observed decreased FA in the preterm compared to full term sample [32–34]. Studies of adolescents and adults born preterm are inconsistent in their findings; studies find areas where preterm groups demonstrate significantly decreased FA [35, 36], increased FA [38], both increased and decreased FA [37, 39, 40], or no differences between the preterm and full term groups [41]. A limitation of voxel-based approaches for group comparisons is that brain images must not only be initially co-registered to a common template based on brain shape and tissue boundaries, but must also be normalized or warped to ensure proper alignment of white matter tracts across subjects [28]. The requirements for normalization may be particularly problematic for comparisons involving clinical populations, such as children born preterm, where individual variability in ventricular size and/or unusual image contrasts are likely to be higher than in typical populations. Such variations can require higher degrees of warping to achieve normalization and may result in the comparison of non-equivalent white matter structures. Imprecise normalization opens the possibility that group differences may reflect partial volume effects rather than real differences in the properties of any specific white matter structure [28].

Diffusion MRI analyzed with tractography is generally regarded as the most precise method for fiber reconstruction within individual subjects [42]. Though prone to similar methodological limitations in terms of initial co-registration for anatomical referencing, tractography methods have been developed that do not require normalization for the comparison of equivalent white matter tracts across subjects [42, 43]. Tracking within native space allows for the comparison of equivalent fiber pathways across individuals even if those tracts are in slightly different locations within the brain. Tractography methods have increasingly been used to identify and interrogate white matter status of specific fiber pathways in children born preterm. The dMRI tractography studies that compare preterm neonates at- and near- term equivalent ages with full term neonates provide evidence of early impaired connectivity [44–46]. Tractography studies also find associations between perinatal diffusion measures and clinical outcomes [7, 16, 44, 47–52] and FA increases with increasing gestational age [51]. Group differences in diffusion properties of white matter in preterm compared to full term samples have also been investigated in older children [9, 10, 12] and adolescents [11, 13]. These structural studies have generally observed decreased mean FA of white matter tracts in the preterm sample compared to the full term sample [9–12]. However, studies have also observed regions of increased FA in the preterm sample [11] or no difference between the groups [13].

Tractography studies of preterm samples have typically focused on analysis of a single fiber tract or a small group of functionally related pathways, possibly because manual tractography approaches are laborious and time-consuming. Recently developed automated approaches for fiber tracking [42, 53] now afford the ability to rapidly identify multiple white matter tracts within individuals of a given sample. An advantage of the Automated Fiber Quantification method (AFQ) [42] is that it identifies equivalent brain structures in native space within each subject individually, and also allows for the comparison of diffusion properties along the trajectory of each tract (tract profiles) rather than a comparison of averaged diffusivity values across the entire tract. Analysis of tract profiles has the advantage of revealing localized group differences that might otherwise be obscured by entire tract means, while also limiting the possibility of including tract regions where partial voluming with gray matter may be more prevalent [11, 42, 54, 55]. In the current study, we compared tract profiles from a sample of children and adolescents born either preterm or full term to determine if group differences would occur throughout periventricular tracts segments and also diffusely in tracts non-adjacent to the lateral ventricles. In addition, we compared tract profiles of the preterm and full term groups to determine whether preterm children would demonstrate increased or decreased FA both within and across different tract trajectories in comparison to full term controls.

Based on the evidence described above, we hypothesized that we would observe group differences in the tracts known to course adjacent to the lateral ventricles: the cortical-spinal tracts, forceps major, forceps minor, inferior fronto-occipital fasciculus, and inferior longitudinal fasciculus [56]. We anticipated that the preterm group would demonstrate both decreased and increased FA compared to the full term group within these tracts, based on previous findings in a voxel-based analysis of this cohort [38] and on recent tractography results in children and adolescents born preterm [11]. We explored evidence for diffuse white matter impairments by examining evidence for group differences in FA within tracts that do not course adjacent to the lateral ventricles: the arcuate fasciculus, uncinate fasciculus, anterior thalamic radiations, cingulum and anterior superior longitudinal fasciculus. In general, group differences identified in this age range were expected to represent the long-term consequences of white matter injury and/or dysmaturity that may occur after preterm birth.

## Materials and Methods

### Participants

Participants took part in a multiple site study of prematurity outcomes [38, 57–61]. The current paper reports on data from participants who enrolled in the Palo Alto, CA site of that study and completed MRI scanning at Stanford University. DTI data from a subset of the preterm group was previously analyzed using different analytic approaches [38, 60, 61]. DTI data from the full term group was previously included in a larger normative sample analyzed using tractography [42]. Preterm birth was defined as gestational age < 36 weeks. Range of current preterm sample was 26.0–34.5 weeks. Full term was defined as gestational age ≥ 37 weeks. Range of current full term sample was 37.0–40.0 weeks. Exclusion criteria for all participants included active seizure disorder, hydrocephalus, receptive vocabulary score < 70, sensorineural hearing loss, and non-native speaker of English. Approval for the study was granted by the Stanford University institutional review board #IRB-6985. A parent or legal guardian provided informed written consent and children provided written assent. Participants were compensated for participation.

Participants were between the ages of 9–17 years old at the time of scanning (n = 46; 23 males, mean age = 12.9 ±2.2), and were born either preterm (n = 28) or full term (n = 20). We excluded from analysis one child born preterm who had no arcuate fasciculus bilaterally, had very abnormal values of FA throughout the brain, and who was described in a case study [62], and one child born full term with an incidental finding of an arachnoid cyst. Thus, the final number of participants analyzed for the preterm group was 27 and for the full term group 19. Full term participants were evaluated and scanned for research purposes only.

Demographic data for the preterm and full term groups is presented in Table 1. The groups did not differ significantly in age, gender, handedness, maternal education, and non-verbal or verbal IQ. [38, 57–61]. Maternal education level was dichotomized with “low” defined as less than a college degree and “high” as at least a college degree. IQ was measured by the Wechsler Abbreviated Scale of Intelligence (WASI), a widely used, nationally standardized test of general intellectual ability [63]. By design, the preterm sample had significantly decreased gestational age and birth weight than the full term sample. One full term and three preterm subjects were left-handed, and three full term and two preterm subjects were ambidextrous, as measured by the Edinburgh Handedness Inventory [64]. Review of T1 images revealed that 4 of the 27 preterm subjects had extreme ventricular enlargement that might affect measurement of diffusion properties in the periventricular regions [55]. The analyses described below were repeated excluding these subjects to assure that the reported group differences were not driven by their data.

**Table 1:** Demographic Measures: Preterm and Full Term Groups.

	Preterm (*n* = 27) M ± SD or *n* (%)	Full Term (*n* = 19) M ± SD or *n* (%)	*t* or *X* ^*2*^
Age	12.92 ± 2.32	12.90 ± 2.12	-0.03
Males	14 (52%)	9 (47%)	0.09
Maternal Education	3 (11%)	6 (32%)	2.97
White	18 (67%)	10 (53%)	0.92
Non-Verbal IQ	106.63 ± 14.37	107.26 ± 12.92	0.88
Verbal IQ	108.82±16.74	113.63±16.74	0.35
Handedness	62.31 ± 57.64	60.53 ± 42.23	-0.11
Gestational Age (weeks)	28.31 ± 2.31	39.17 ± 1.13	18.89**
Birth Weight (g)	1198 ± 465	3154 ± 407	14.76**

**p < .01

Medical information was available for 25 of 27 preterm subjects. Medical complications at birth in the preterm group were: six with abnormal findings on head ultrasounds or MRIs (≥ grade 2 intraventricular hemorrhage, echodensities, or cystic lesions), two with mildly abnormal findings (grade 1 hemorrhage or choroid plexus cyst); 12 had respiratory distress syndrome, five developed bronchopulmonary dysplasia (BPD) or chronic lung disease; four had patent ductus arteriosus (PDA); none had necrotizing enterocolitis (NEC); one was small for gestational age (≤ 3rd percentile birth weight for gestational age).

A neuroradiologist unaware of the child’s medical conditions evaluated the T1- and T2-weighted MRI scans of the preterm group, using a white matter scoring system derived from [65]. Each scan was scored from 5 to 15, based on a 3-point scale for five white matter features: white matter signal abnormality, periventricular white matter volume loss, cystic abnormalities, ventricular dilation, and thinning of the corpus callosum. We classified the scans as normal (score ≤6), mild-moderate (>6 and ≤12), or severe (>12) injury. Of the 27 preterm subjects, 13 were classified as normal, 10 were classified as having mild-moderate injury, and 4 as having severe injury. Previous evidence has shown that dMRI can be sensitive to microstructural differences in white matter in the absence of gross structural injuries in preterm neonates [66] and adolescents born preterm [11]. Moreover, a study of white matter volumes found that the results of both early and concurrent MRI scans did not affect white matter measurements [67]. We included all preterm subjects in group analyses regardless of gross white matter injury scores, consistent with other studies of this population [11]. However, we analyzed the data with and without the children with severe injury included in the group (see below).

### MRI Acquisition

MRI data were acquired on a 3T Signa Excite scanner (GE Medical Systems, Milwaukee, WI) at Stanford University. T1 images included three high resolution inversion recovery (IR)-prep 3D fast-spoiled gradient (FSPGR) scans collected in the axial plane. The first T1 (field of view (FOV) = 240 x156 mm, matrix size = 256 x 192, voxel size 0.9375 x 0.8125 x 1.2 mm, TI = 300 ms, flip angle = 15 degrees, number of excitations (NEX) = 1) was subsequently averaged with two additional T1 scans (FOV = 240 x 180 mm, matrix size = 260 x 192, voxel size 0.9231 x 0.9375 x 0.9 mm, TI = 300 ms, flip angle = 15 degrees, NEX = 1). T2 fluid attenuated inversion recovery (FLAIR) images were also collected (repetition time 9500ms, echo time 120ms, FOV 220, slices 5.0mm, no gaps) but were not analyzed.

For dMRI and tractography, a diffusion-weighted, single-shot, spin-echo, echo-planar imaging sequence (TE = 80 ms, TR = 6500 ms, FOV = 240 mm, matrix size = 128 x 128, voxel size = 1.875 x 1.875 x 2 mm) was used to acquire 60 slices, in 30 different diffusion directions (b = 900). The sequence was repeated 4 times for improved SNR, and 10 non-diffusion weighted (b = 0) volumes were collected as well.

### Data Preprocessing

The T1 images were co-registered to each other using a mutual information maximization algorithm (SPM5, http://www.fil.ion.ucl.ac.uk/spm/) and subsequently averaged for improved contrast. A trained research assistant manually identified the anterior and posterior commissures on the midsagittal plane, and these points were used to align the averaged anatomical image to a canonical ac-pc orientation, using a rigid body transformation (implemented in SPM5; no warping was applied).

Diffusion MR images were pre-processed with open-source software, mrDiffusion (http://white.stanford.edu/newlm/index.php/MrDiffusion) implemented in MATLAB R2012a (Mathworks, Natick, MA). Eddy current distortions and subject motion in the diffusion weighted images were corrected by a 14-parameter constrained non-linear co-registration algorithm based on the expected pattern of eddy-current distortions, given the phase-encoding direction of the acquired data [68]. No subjects were excluded due to excessive head motion and no volumes were discarded. Additional analyses confirmed that full term and preterm groups also did not significantly differ in terms of translational head motion detected during image preprocessing (see S1 Text). Diffusion data were aligned to the T1 anatomical scans that had been averaged and rotated to align with the ac-pc plane. Alignment between dMRI and T1 data was achieved by registering the b0 images to the resampled T1 image using the same mutual information maximization algorithm used for T1 image co-registration provided through SPM5.

For each voxel in the aligned and resampled volume, tensors were fit to the diffusion measurements using a robust least-squares algorithm designed to remove outliers and data points corrupted by motion at the tensor estimation step (Robust Estimation of Tensors by Outlier Rejection (RESTORE) [69]. A continuous tensor field was estimated using trilinear interpolation of the tensor elements. We computed the eigenvalue decomposition of the diffusion tensor and the resulting three eigenvalues (λ1, λ2, λ3) were used to compute fractional anisotropy (FA), axial diffusivity (AD), and radial diffusivity (RD) [19]. The accuracy of image co-registration was verified in individual subjects by overlaying a white matter mask that included diffusion image voxels with FA >0.2 onto the average T1 image and visually inspecting the anatomical alignment.

### Fiber tracking and segmentation

Fiber tracking and tract segmentation were performed using an open source software, Automated Fiber Quantification (AFQ; https://github.jyeatman/AFQ) implemented in MATLAB R2012a (Mathworks, Natick, MA).

AFQ consists of three major processing steps: (1) whole-brain tractography (2) automatic tract segmentation and cleaning, and (3) fiber quantification [42]. Whole brain tractography was performed using a deterministic streamlines tracking algorithm (STT) [69–71], with a fourth-order Runge-Kutta path integration method [72]. The fiber tracking algorithm was seeded with a white matter mask defined as all the voxels with FA value greater than 0.2 in the entire brain volume. Tracking proceeded in all directions and stopped when FA dropped below 0.15 or when the angle between the extension of a line in the direction of the current step and the direction of the next step was greater than 30°.

Tract segmentation was achieved using a multiple waypoint ROI procedure as defined by [73] and automated in AFQ [42]. During AFQ processing, an estimated non-linear transformation [74] was applied to automatically warp predefined ROIs from the Montreal Neurological Institute (MNI) template into an individual’s native space. In this approach, ROIs are defined such that they isolate the central portion of the tract where fibers are most coherently bundled. Fibers are considered to belong to a specific tract only if they pass through both waypoint ROIs as specified in [73]. Using this procedure, we isolated for each individual 18 major pathways in the participant’s native space. This included the posterior and anterior segments of the corpus callosum (forceps major, FMajor and forceps minor, FMinor, respectively), and 8 pairs of bilateral pathways: the anterior thalamic radiations (ATR), corticospinal tract (CST), cingulum (Cing), inferior fronto-occipital fasciculus (IFOF), inferior longitudinal fasciculus (ILF), anterior superior longitudinal fasciculus (aSLF), uncinate fasciculus (UF) and the arcuate fasciculus (Arc). Tracts were cleaned automatically using a statistical outlier rejection algorithm [69] for removing outlier fibers.

In a small proportion of tracts and individuals, cleaned tracts still included looping fibers that re-crossed both ROIs: Cing-L (4 preterm subjects; 1 of 4 had enlarged ventricles); FMajor (1 preterm subject; 0 of 1 had enlarged ventricles); aSLF-L (1 full term subject). These fibers were removed using Quench, a gesture based segmentation and visualization tool (http://white.stanford.edu/newlm/index.php/QUENCH). No other tracts required manual editing. The same investigator (KET) also verified that ROI placement for fiber segmentation was anatomically accurate in the small proportion of subjects in whom tracts were not reliably identified (see S1 Table). This procedure ensured that failure to detect these tracts was unlikely to be a consequence of inaccurate ROI placement.

### Fiber Tract Quantification

FA was calculated at 30 equidistant nodes along a central portion of each fiber tract bounded by the same two ROIs used for tract segmentation. Tract extremities beyond these ROIs were not included in the analysis. This procedure generates, for every tract and every individual, an FA tract profile that describes the variations in FA along the central portion of the tract. At each node, diffusion properties (FA, RD, AD, MD) were calculated by taking a weighted average across all fibers belonging to this tract. Each fiber’s contribution to the average was weighted by the probability that a fiber was a member of the fascicle, computed as the Mahalanobis distance from the tract core [42]. This procedure minimizes the contribution of fibers located further from the fiber tract core that are more likely to reflect a mixture of gray and white matter or of different tracts, and so minimizes the effect of partial voluming on diffusion estimates.

### Classification of periventricular tracts and nodes

Tracts were first classified as periventricular or non-periventricular based on a white matter atlas [56]. A tract was considered to be periventricular if its trajectory traverses adjacent to the lateral ventricles. Based on this definition, we classified the CST, FMajor, FMinor, IFOF, and ILF as periventricular tracts and the Arc, UF, ATR, Cing and aSLF as non-periventricular tracts. We next loaded tract renderings of each of the periventricular tracts from representative full term and preterm subjects in Quench, to visually approximate the location of the lateral ventricles and to confirm that the ROI bounded portion of the tract traverses adjacent to the lateral ventricles. Nodes that were adjacent to the lateral ventricles were approximated on tract renderings using ac-pc coordinate information provided for each node in the tract profile.

### Volume estimation

Volume estimates were obtained for the core region of each tract (ie., region between ROI1 and ROI2) and the whole-brain fiber group for each subject. Volume was estimated as the number of voxels covered by one or more streamlines in the aligned T1-image [75]. Volume estimates for each tract were normalized by dividing each tract volume estimate by the volume estimate for the whole brain fiber group. This step was performed to confirm that differences in tract diffusion properties did not reflect a general difference in white matter volume between full term and preterm groups.

### Statistical Analyses

#### Analyses of demographic variables

Chi-square tests and two-tailed *t*-tests for independent samples were used to examine differences between the preterm and full term samples on demographic variables.

#### Group comparisons of tract properties: omnibus tests

Given that white matter changes are known to occur during the age range of the current sample [76], we first assessed FA in relation to age in the preterm and full term groups separately to determine whether it was necessary to control for age in group analyses. This step was achieved by calculating Spearman correlations between age at DTI scan and mean FA for each tract. In the full term group, we found three tracts (Arc-R, Cing-L, Cing-R) in which age associations were present. In the preterm group, we found an age association within the ILF-L. The resulting statistics of this analysis are presented in S2 Table. To identify age-associations that may have been obscured by whole tract mean measures, we also calculated Spearman correlations between age at DTI scan and FA at 30 locations along each tract. These analyses revealed additional significant positive age-associations (p < 0.05, uncorrected) within the full term (CST-L, CST-R, ATR-L, ATR-R,) and preterm groups (CST-R, FMinor, UF-R, IFOF-R) that were not apparent in analyses with whole tract mean measures. Given these findings, we included participant’s age at the time of scanning in the subsequent ANCOVAs as a continuous covariate in order to isolate group differences independent of age. For consistency, we include age at DTI scanning as a continuous covariate for all tracts.

We assessed group differences in tract properties by calculating, for each tract, a mixed design three-way analysis of co-variance (ANCOVA) [77], with Group (preterm versus full term) as a between-subject factor, while Hemisphere (left versus right tract homologs) and Location (30 nodes along the tract) served as within-subject measures. FA profiles of the Forceps Major and Forceps Minor were divided along the midsagittal plane to allow a comparison of their left (nodes 1–15) and right (nodes 16–30) sections, similarly to the left and right homologs of each tract. Degrees of freedom were corrected using Greenhouse-Geisser estimates in cases where sphericity was violated [78]. Group differences were considered to be significant at p < 0.05. To control for multiple comparisons across tracts, the significance of omnibus tests was determined using a false discovery rate (FDR) of 5% [79].

To ensure that the results of this analysis were not driven by the four preterm subjects with extreme ventricular enlargement, ANCOVA analyses were repeated without these subjects. To determine if the present pattern for group differences in FA were secondary to differences in volume, we calculated t-tests for independent samples to examine whether mean volume for all tracts and the whole brain fiber group systematically varied between preterm and full term groups. T-tests for independent samples were then repeated using normalized tract volume for those tracts found to exhibit significant group differences.

Finally, since previous studies of older preterm children and adolescents have reported group differences in MD [11, 40], we also repeated ANCOVA analyses with mean diffusivity (MD) for completeness. Spearman correlations calculated between age at DTI scan and mean tract MD revealed significant age associations (p<0.05) in 9 out of 18 tracts in the full term group (S3 Table). In the preterm group a significant age-association was observed only for the IFOF-R (S3 Table). Based on these findings, we included age at DTI scan as a continuous covariate to isolate group differences with MD that were independent of age. For consistency, we included age at DTI scanning as a continuous covariate for all tracts. The significance of omnibus tests with measures of MD was determined using a false discovery rate (FDR) of 5% [79].

#### Location- and hemisphere- specific contrasts

To identify the hemisphere and tract segments (nodes) responsible for the group differences identified by the omnibus tests, we followed up on significant ANCOVA effects with sets of two-tailed t-tests for independent samples, comparing the FA values of the preterm and full term groups at each node along the tract profile (separated by hemisphere). These comparisons were calculated only for tracts identified by the ANCOVAs such that: (1) the tract showed a significant main effect of Group, or (2) the tract showed a significant interaction between Group and Location, or (3) the tract showed a significant interaction between Group and Hemisphere. We considered (2) and (3) essential because group effects could have occurred in opposing directions within different segments of the same tract or in different hemispheric homologs of the same tract. Location and hemisphere specific contrasts were not investigated further if group differences did not survive correction for multiple comparisons across tracts.

We employed a nonparametric permutation-based method to control for the 30 comparisons along the tract [80]. This procedure produced a family-wise error corrected cluster size and a critical t-value for each of the candidate tracts. Tract segments were considered significant if differences occurred either (1) in a sufficient number of adjacent nodes to meet the criteria for a family-wise error corrected cluster size or (2) in nodes in which the effect size was greater than the critical t-value.

#### Exploratory analyses: gestational age, AD and RD

To further interrogate group differences, we analyzed the association between individual gestational age at birth in the preterm group and FA within tract segments that demonstrated significant group differences. We calculated Spearman correlations between gestational age at birth and mean FA extracted from the segments where significant group differences were detected in the along tract profile comparisons. These analyses were limited to the preterm group, to avoid a pseudo-correlation reflecting the group differences in both variables. To investigate the contributions of AD and RD to group differences in FA values, we computed a separate one-way multivariate analysis of variance (MANOVA) for each cluster of nodes identified in the tract profile analysis as showing a significant group difference in FA. Group (preterm versus full term) served as the between-subject variable and mean AD and mean RD served as the dependent variables. We chose to enter AD and RD into the same model in order to reduce the number of comparisons and increase power for detecting group differences.

## Results

### Fiber Tracking

**Fig 1:**
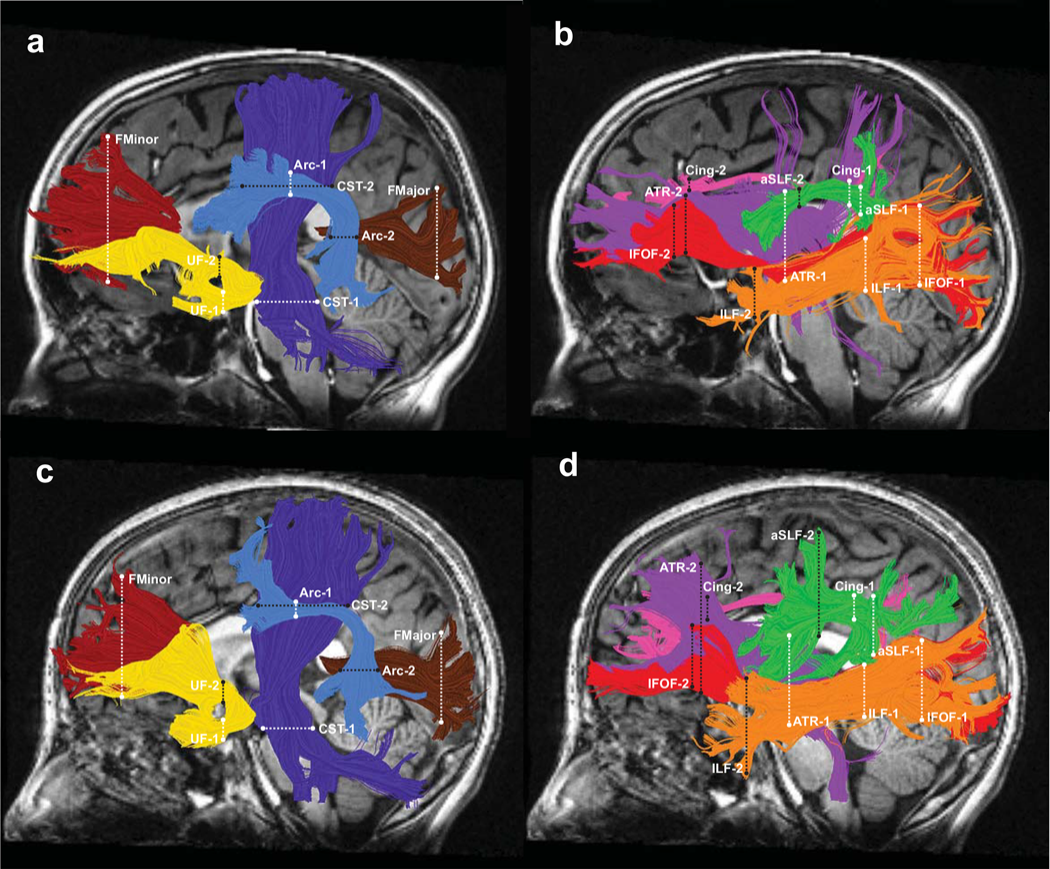
Tractography of 18 major cerebral white matter tracts. The left hemispheric cerebral tracts are displayed on mid-sagittal T1 images from a representative full term subject (a, b) and a representative preterm subject (c, d). Right hemisphere tract renderings not shown. Panels a and c illustrate the following tracts: Arcuate Fasciculus (Arc) = light blue; Corticospinal Tract (CST) = dark blue; Forceps Major (FMajor) = brown; Forceps Minor (FMinor) = dark red; Uncinate Fasciculus (UF) = yellow. Panels b and d illustrate the following tracts: Anterior Thalamic Radiation (ATR) = purple; Cingulum (Cing) = magenta; Inferior Fronto-occipital Fasciculus (IFOF) = red; Inferior Longitudinal Fasciculus (ILF) = orange; Anterior Superior Longitudinal Fasciculus (aSLF) = green. Dashed lines represent the location of the regions of interest (ROIs) used to isolate each cerebral tract; ROI 1, white; ROI 2, black.

Using automated fiber segmentation procedures, we successfully identified 18 cerebral tracts of interest in the majority of both full term and preterm individuals (Fig 1). A small number of tracts were missed in one to three subjects (see S1 Table). In addition, we were unable to identify the Arc-R in 6 full term and 4 preterm subjects. We attribute the difficultly in identifying the Arc-R to limitations of deterministic tractography approaches that cannot account for higher tract curvature and increased partial voluming with the aSLF-R, a finding consistent with several other reports [43, 81–83].

**Fig 2:**
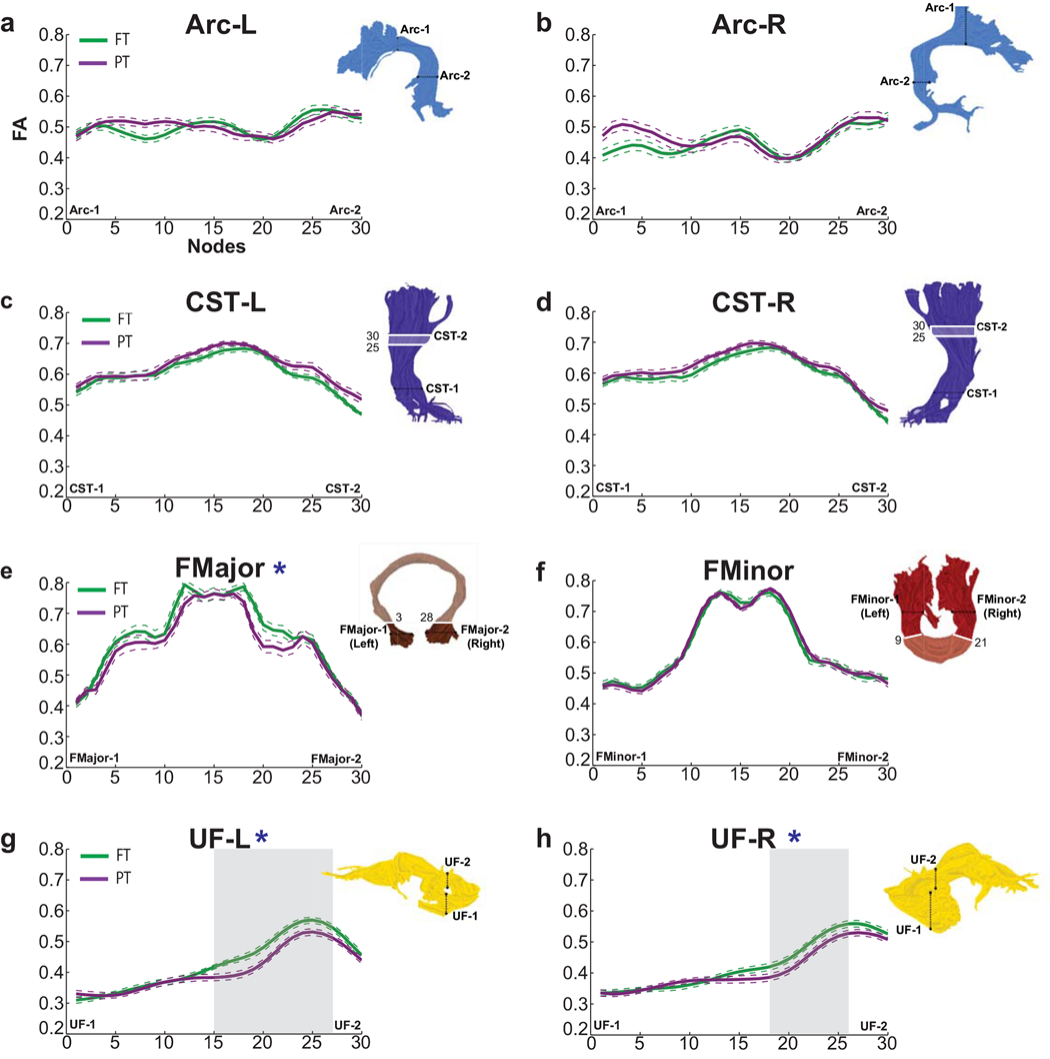
Tract profiles in the preterm and full term groups. Mean FA profiles are shown for each of the cerebral tracts depicted in Fig 1A and 1C, for the full term group (solid green line) and the preterm group (solid purple line). Visualized tract profiles: left (a) and right (b) arcuate fasciculus; left (c) and right (d) corticospinal tract; (e) forceps major and (f) forceps minor; and left (g) and right (h) uncinate fasciculus. FA values are plotted for 30 equidistant nodes between the two ROIs used to isolate the core of each tract. For all tracts, node 0 corresponds to ROI-1 and node 30 corresponds to ROI-2. Dashed lines indicate ± 1 standard error of the mean. Tracts with significant Group by Location interactions in the omnibus test are indicated with a blue *. Shaded gray background indicates a tract segment where mean FA of the PT is significantly decreased compared to the FT group. Tract renderings from a representative full term subject pictured in Fig 1A and 1B are shown next to each profile for illustration purposes. Light shading and white outline on the tract rendering indicates the approximate location of the periventricular zone in this participant, and the node numbers defining this zone are indicated next to the rendering. Arc = Arcuate Fasciculus; CST = Corticospinal Tract; FMajor = Forceps Major; FMinor = Forceps Minor; UF = Uncinate Fasciculus; PT = Preterm; FT = Full term; FA = Fractional Anisotropy; L = Left; R = Right.

**Fig 3:**
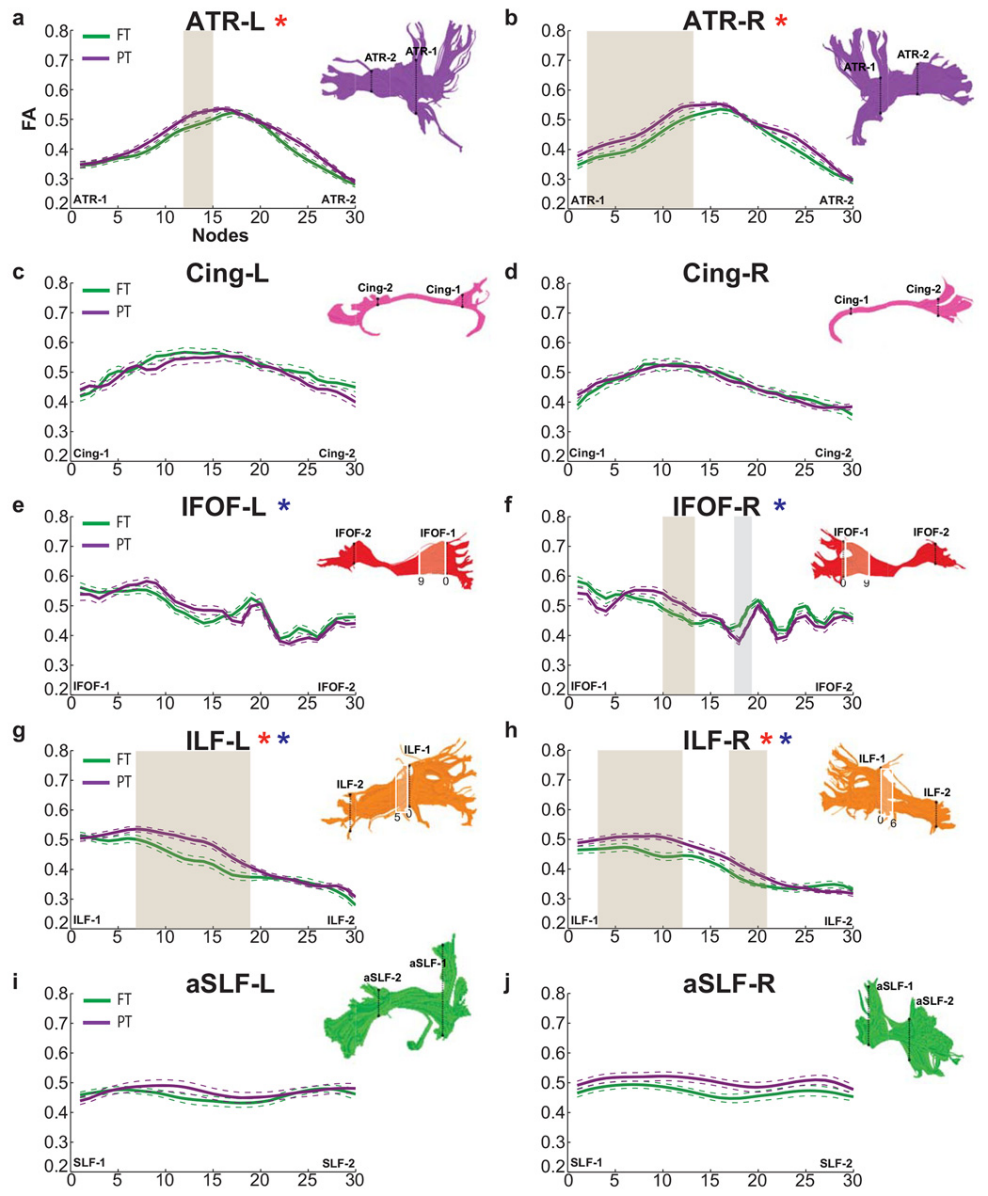
Tract profiles in the preterm and full term groups (continued). Color coding and visualization scheme same as in Fig 2 (see caption to Fig 2). Visualized tract profiles: left (a) and right (b) anterior thalamic radiations; left (c) and right (d) cingulum; left (e) and right (f) inferior fronto-occipital fasciculus; left (g) and right (h) inferior longitudinal fasciculus; and left (i) and right (j) anterior superior longitudinal fasciculus. Dashed lines indicate ± 1 standard error of the mean. FA values are plotted for 30 equidistant nodes between two ROIs used to isolate the core of each tract. For all tracts Node 0 corresponds to ROI-1 and Node 30 corresponds to ROI-2. Tracts with significant main effect of Group in the omnibus test are indicated with a red *. Tracts with significant Group by Location interactions in the omnibus test are indicated with a blue *. Shaded gray regions indicate tract segments where mean FA of the PT group is significantly decreased than the FT group. Shaded light brown regions indicate tract segments where mean FA of the FT group is significantly increased than the PT group. Tract renderings from a representative full term subject pictured in Fig 1A and 1B are shown next to each profile for illustration purposes. Light shading and white outline on the tract rendering indicates the approximate location of the periventricular zone in this participant, and the node numbers defining this zone are indicated next to the rendering. ATR = Anterior Thalamic Radiation; Cing = Cingulum; IFOF = Inferior Fronto-occipital Fasciculus; ILF = Inferior Longitudinal Fasciculus; aSLF = Anterior Superior Longitudinal Fasciculus; PT = Preterm; FT = Full term; FA = Fractional Anisotropy; L = Left; R = Right.

Mean FA profiles from the preterm and full term groups for all 18 tracts are presented in Figs 2 and 3. Tracts are separated into two figures for visualization purposes. FA profiles presented in Fig 2 correspond to the tracts shown in Fig 1A and 1C. FA profiles in Fig 3 correspond to the tracts shown in Fig 1B and 1D. Visual inspection of the suspected periventricular tracts (CST, FMajor, FMinor, IFOF and ILF) in representative full term and preterm subjects confirmed that these tracts contained regions adjacent to the lateral ventricles. These periventricular regions are marked by light shading on the relevant tract renderings in Figs 2 and 3. Tract results are first described based on the order in which they appear in Figs 2 and 3 (Table 2). For comparative purposes, group mean tract FA values for all 18 cerebral tracts are presented in Table 3. In subsequent sections, this same ordering is then sub-divided by the direction of group differences: Preterm < Full term and Preterm > Full term (Tables 4 and 5).

**Table 2:** ANCOVA results for the comparison of preterm and full term FA profiles.

Tract	Main Effect of Group	Group by Location Interaction	Group by Hemisphere Interaction
Arc	F = 0.56, p = 0.46	F = 1.87, p = 0.09	F = 0.49, p = 0.49
CST	F = 2.93, p = 0.09	F = 0.69, p = 0.53	F = 0.02, p = 0.89
FMajor	F = 2.92, p = 0.10	F = 2.43, p = 0.04*	F = 0.41, p = 0.84
FMinor	F = 0.01, p = 0.91	F = 1.12, p = 0.35	F = 2.71, p = 0.12
UF	F = 4.27, p = 0.05	F = 3.87, p < 0.01**	F = 0.49, p = 0.49
ATR	F = 6.16, p = 0.02*	F = 1.52, p = 0.20	F = 0.72, p = 0.40
Cing	F = 0.34, p = 0.57	F = 1.09, p = 0.37	F = 1.06, p = 0.31
IFOF	F = 0.16, p = 0.69	F = 4.02, p < 0.01**	F = 0.48, p = 0.49
ILF	F = 13.66, p < 0.01**	F = 5.24, p < 0.01**	F = 0.18, p = 0.67
aSLF	F = 3.24, p = 0.08	F = 1.13, p = 0.34	F = 1.15, p = 0.29

*p < 0.05

**p < 0.01

Arc = Arcuate Fasciculus; CST = Corticospinal Tract; FMajor = Forceps Major; FMinor = Forceps Minor; UF = Uncinate Fasciculus; ATR = Anterior Thalamic Radiation; Cing = Cingulum; IFOF = Inferior Fronto-occipital Fasciculus; ILF = Inferior Longitudinal Fasciculus; aSLF = Anterior Superior Longitudinal Fasciculus

**Table 3:** Mean Tract FA of 18 cerebral white matter tracts in Preterm and Full Term groups.

Tract	Preterm Mean FA (stdev)	Full Term Mean FA (stdev)
Arc		
Left	0.51 (0.04)	0.50 (0.03)
Right	0.47 (0.04)	0.45 (0.04)
CST		
Left	0.62 (0.03)	0.60 (0.03)
Right	0.62 (0.04)	0.60 (0.03)
FMajor		
Left	0.61 (0.07)	0.63 (0.06)
Right	0.58 (0.06)	0.61 (0.04)
FMinor		
Left	0.57 (0.03)	0.57 (0.03)
Right	0.60 (0.03)	0.59 (0.03)
UF*		
Left^+^	0.41 (0.03)	0.43 (0.03)
Right^+^	0.41 (0.04)	0.43 (0.03)
ATR*		
Left^+^	0.43 (0.03)	0.41 (0.03)
Right^+^	0.45 (0.05)	0.43 (0.03)
Cing		
Left	0.50 (0.06)	0.51 (0.04)
Right	0.46 (0.04)	0.46 (0.05)
IFOF*		
Left	0.48 (0.04)	0.48 (0.03)
Right^+^	0.48 (0.03)	0.48 (0.02)
ILF*		
Left^+^	0.44 (0.03)	0.41 (0.03)
Right^+^	0.43 (0.03)	0.40 (0.03)
aSLF		
Left	0.47 (0.07)	0.46 (0.03)
Right	0.50 (0.06)	0.47 (0.05)

stdev = standard deviation; Arc = Arcuate Fasciculus; CST = Corticospinal Tract; FMajor = Forceps Major; FMinor = Forceps Minor; UF = Uncinate Fasciculus; ATR = Anterior Thalamic Radiation; Cing = Cingulum; IFOF = Inferior Fronto-occipital Fasciculus; ILF = Inferior Longitudinal Fasciculus; aSLF = Anterior Superior Longitudinal Fasciculus

* indicates tracts that demonstrated significant group differences in omnibus tests after correction for comparisons across tracts

+ indicates tracts that contributed to group difference identified with omnibus tests

**Table 4:** Locations along the white matter tracts in which Preterm and Full Term groups showed significant group differences in Fractional Anisotropy.

Tract	Preterm Mean FA of significant nodes (95% CI)	Full Term Mean FA of significant nodes (95% CI)	*t* statistic for mean FA of significant nodes	Correlation with Gestational Age (*rs*)
PT Mean FA < FT Mean FA				
UF				
Left				
15–27^a^ ^,^ ^b^	0.46 (0.44–0.48)	0.50 (0.48–0.52)	-3.19	0.31
Right				
18–26^b^	0.46 (0.43–0.48)	0.49 (0.47–0.51)	-2.38	0.20
IFOF				
Right				
18–19^a^	0.41 (0.39–0.43)	0.46 (0.44–0.49)	-3.80	0.39*
PT Mean FA > FT Mean FA				
ATR				
Left				
12–15^a^	0.52 (0.50–0.53)	0.48 (0.46–0.51)	2.84	-0.11
Right				
2–13^b^	0.46 (0.44–0.48)	0.43 (0.41–0.44)	2.73	-0.12
IFOF				
Right				
10–13^a^	0.51 (0.49–0.54)	0.47 (0.45–0.49)	2.98	-0.13
ILF				
Left				
7–19^a^ ^,^ ^b^	0.49 (0.47–0.51)	0.43 (0.41–0.46)	3.60	-0.12
Right				
4–12^a^ ^,^ ^b^	0.50 (0.49–0.52)	0.46 (0.44–0.48)	3.40	-0.35
17–21^a^	0.40 (0.38–0.42)	0.36 (0.34–0.38)	2.94	-0.07

^a^ significant for *t*-value, corrected

^b^ significant for cluster size, corrected

* p < 0.05 for Spearman correlation

CI = confidence interval; rs = Spearman correlation coefficient; PT = Preterm; FT = Full Term; FMajor = Forceps Major; UF = Uncinate Fasciculus; IFOF = Inferior Frontal Occipital Fasciculus; ATR = Anterior Thalamic Radiation; ILF = Inferior Longitudinal Fasciculus.

**Table 5:** Mean AD and RD within segments of the white matter tracts in which group differences in FA reached statistical significance.

Tract (Significant Cluster)	Preterm Mean of cluster (95% CI)	Full Term Mean of cluster (95% CI)	F
PT Mean FA < FT Mean FA			
UF-L (15–27)			
AD	1.35 (1.32–1.37)	1.28 (1.26–1.30)	17.79**
RD	0.57 (0.55–0.59)	0.59 (0.58–0.61)	2.55
UF-R (18–26)			
AD	1.32 (1.29–1.35)	1.27 (1.25–1.29)	8.51**
RD	0.57 (0.55–0.59)	0.59 (0.58–0.61)	3.03
IFOF-R (18–19)			
AD	1.32 (1.29–1.35)	1.26 (1.23–1.28)	11.61**
RD	0.60 (0.58–0.63)	0.64 (0.63–0.66)	8.53**
PT Mean FA > FT Mean FA			
ATR-L (12–15)			
AD	1.30 (1.27–1.34)	1.32 (1.29–1.35)	0.62
RD	0.57 (0.54–0.59)	0.54 (0.52–0.55)	5.71*
ATR-R (2–13)			
AD	1.24 (1.21–1.27)	1.24 (1.21–1.26)	0.04
RD	0.61 (0.58–0.63)	0.57 (0.54–0.59)	7.35**
IFOF-R (10–13)			
AD	1.38 (1.35–1.40)	1.42 (1.40–1.45)	7.97**
RD	0.65 (0.63–0.68)	0.61 (0.58–0.63)	6.58*
ILF-L (7–19)			
AD	1.34 (1.30–1.38)	1.35 (1.31–1.38)	0.48
RD	0.67 (0.65–0.70)	0.61 (0.59–0.63)	19.60**
ILF-R (4–12)			
AD	1.34 (1.32–1.37)	1.41 (1.37–1.45)	7.08*
RD	0.64 (0.62–0.66)	0.61 (0.59–0.63)	3.84
ILF-R (17–21)			
AD	1.23 (1.20–1.26)	1.24 (1.22–1.26)	0.13
RD	0.72 (0.70–0.74)	0.68 (0.65–0.70)	6.12*

* *p* < .05

** *p* < .01

CI = Confidence Interval; PT = Preterm; FT = Full Term; FMajor = Forceps Major; UF = Uncinate Fasciculus; IFOF = Inferior Fronto-occipital Fasciculus; ATR = Anterior Thalamic Radiation; ILF = Inferior Longitudinal Fasciculus; L = left; R = right.

### Group comparisons of tract properties: Omnibus Tests (FA)

ANCOVAs calculated using measures for FA and age as a covariate for each tract revealed significant main effects of Group in the ATR and ILF (Table 2). Significant interactions were found between Group and Location in the FMajor, UF, IFOF, and ILF. Group comparisons remained significant after controlling for multiple comparisons across tracts at a 5% criterion for FDR for all tracts except for the FMajor. For this reason, location and hemisphere specific contrasts were not investigated further for the FMajor. No tracts demonstrated significant Group by Hemisphere interaction effects. No significant group effects or interactions were observed for the following five tracts: Arc, CST, FMinor, Cing and aSLF. In addition, no tracts demonstrated significant main effects or interactions with age except for a significant Age by Location interaction effect in the cingulum F(4.91,206.3) = 4.02 p < 0.002.

Post-hoc analyses confirmed that group effects identified in the omnibus tests remained significant (p < 0.05) after removing the four preterm subjects diagnosed with extreme ventricular enlargement. Since omnibus effects did not appear to be driven by these 4 cases, these subjects were included in all subsequent analyses.

Analyses of tract volume confirmed that group differences in FA were unlikely to be driven by systematic differences in tract volumes or volume of the whole brain fiber group (S4 Table). Tract volume was lower for the preterm than full term group in the FMajor and IFOF-R but no significant tract volume group differences were observed for any of the other 16 cerebral white matter tracts examined or for the whole brain fiber group from which individual tracts had been segmented (S4 Table). These group differences within the FMajor and IFOF-R remained significant (p < 0.05) after controlling for the size of the whole brain fiber group. Further, the full term group was found to have significantly higher tract volume in tracts where the full term group demonstrated both significantly increased and decreased FA compared on the preterm group (S4 Table). Specifically, within the FMajor, the full term group demonstrated significantly (p < 0.05) higher tract volume than the preterm group. In the IFOF-R, the full term group demonstrated significantly (p < 0.05) higher tract volume than the preterm group, though the full term group was observed to demonstrate regions of both higher and lower FA.

### Group comparisons of tract properties: Omnibus Tests (MD)

ANCOVAs calculated using measures for MD and age as a covariate revealed a significant main effect of Group in the ILF (S5 Table). Significant interactions were found between Group and Location in the FMajor and FMinor (S5 Table). No tracts demonstrated significant Group by Hemisphere interaction effects. A significant main effect of age was observed for the UF (F(1,43) = 4.86, p = 0.03) and significant Age by Location interaction effects were observed for the CST (F(3.09,129.73) = 3.14, p = 0.02), FMinor (F(2.37,101.73) = 4.91, p = 0.006), and UF (F(2.55,109.84) = 2.96, p = 0.04). Group comparisons were no longer significant after controlling for multiple comparisons across tracts at a 5% criterion for FDR. For this reason, location and hemisphere specific contrasts were not investigated further.

### Location- and Hemisphere- specific contrasts (FA)

In each tract that showed a significant main effect of Group or a significant interaction with Group, we compared the mean FA values for the two groups at each node along the tract, using two-tailed t-tests. Tract segments showing significant group differences are indicated in Figs 2 and 3 with a light gray background shading (preterm < full term) or a light brown background shading (preterm > full term). The pattern of results was generally similar in the left and right hemispheres, though the differences did not always rise to the level of statistical significance in both hemispheres. FA was significantly decreased in the preterm group compared to the full term group in the UF bilaterally (Fig 2G and 2H) and anterior segments of the IFOF-R (Fig 3F); see Table 4 for detailed statistics. In contrast, FA was significantly increased in the preterm group compared to the full term group within the ATR bilaterally (Fig 3A and 3B), posterior segments of the IFOF-R (Fig 3F), and the ILF bilaterally (Fig 3G and 3H) (Table 4). In general, these detailed contrasts revealed that the preterm group demonstrated significant decreases as well as significant increases in FA relative to the full term group, in both periventricular and non-periventricular tracts (Figs 2 and 3). Moreover, within specific periventricular tracts (IFOF, ILF), the preterm group demonstrated increased and decreased FA values both within and beyond the periventricular zone (Figs 2 and 3).

### Associations with Gestational Age within the Preterm group

We performed Spearman correlations to determine if the gestational age at birth of the preterm sample was associated with FA within tract segments that demonstrated significant group differences. Correlation coefficients for each tract segment are reported in Table 4. This analysis revealed a significant positive association between gestational age and FA in an anterior segment of the IFOF-R only (rs = 0.39, p < 0.05; Table 4, S1 Fig). No significant associations were observed for the remaining tract segments.

### Group Comparisons: AD and RD

In tract segments in which we detected a significant group difference in FA, we conducted MANOVAs in order to assess whether this difference would be best explained by differences in AD or RD. These analyses revealed significant multivariate effects of group (FT vs PT) for all tracts (p < 0.05). To identify whether group effects identified in the MANOVA were the result of group differences in AD, RD or both, we next performed post-hoc univariate ANCOVAs. The results of this analysis are reported in Table 5. In the three tract segments in which the preterm group had decreased FA compared to the full term group (UF-L, UF-R, and IFOF-R), the preterm group showed increased AD compared to the full term group.

In tract segments in which FA was significantly increased in the preterm group compared to the full term group, the preterm group had significantly decreased RD compared to the full term group in the following tracts: ATR-L, ATR-R, IFOF-R, ILF-L, and ILF-R. The preterm group also had significantly increased AD compared to the full term group, with both AD and RD contributing to increased FA in the IFOF-R and the ILF-R.

## Discussion

### Summary of Results

Using an automated, large-scale tractography approach, we identified 18 cerebral white matter tracts in a sample of children and adolescents born preterm and full term. We found significant group differences in FA within multiple tracts. Compared to the full term controls, the group of children born preterm had decreased FA in the uncinate fasciculus bilaterally and an anterior segment of the right inferior fronto-occipital fasciculus. By contrast, the preterm group had increased FA within the anterior thalamic radiations, posterior segments of the right inferior fronto-occipital fasciculus, and the inferior longitudinal fasciculus. These group differences occurred both within periventricular and non-periventricular tracts segments. Group differences did not appear to be explained by the 4 preterm subjects with ventricular dilation or by individual variations in tract volume. With the exception of the forceps minor, ANCOVA analyses repeated using MD did not reveal evidence for further microstructural differences in tracts beyond those identified using FA. Exploratory analyses revealed a significant positive association between FA and gestational age at birth of the preterm born individuals within an anterior region of the right inferior fronto-occipital fasciculus. Increased FA in the preterm group was generally associated with decreased RD, while decreased FA in the preterm group was associated with increased AD.

### Group differences in periventricular tracts

Consistent with our initial predictions, FA differences between the preterm and full term groups were found within periventricular white matter tracts, including the inferior fronto-occipital fasciculus and the inferior longitudinal fasciculus. Specific contrasts revealed that group differences observed in the right inferior longitudinal fasciculus were found specifically in the periventricular zones within these tracts. These findings are generally consistent with the pathological characteristics of preterm brain injury [16]. They are also consistent with evidence from animal models of prematurity that document the vulnerability of periventricular white matter to injury from hypoxic-ischemic and inflammatory responses experienced during late gestation [84, 85]. In contrast, group differences observed in the right inferior fronto-occipital fasciculus and left inferior longitudinal fasciculus localized to segments that were not directly adjacent to the lateral ventricles. More research is needed to determine whether group differences observed in regions distal to the lateral ventricles reflect white matter injuries or dsymaturation arising from similar or distinct neuropathological processes from those observed in periventricular areas. Such studies may benefit from the use of animal models of prematurity [86] or MR spectroscopy for distinguishing amongst cellular mechanisms related to axonal dysmaturity or astrogliosis that may contribute to white matter injuries associated with prematurity [87].

Contrary to the initial hypothesis, FA of the periventricular left or right corticospinal tracts and forceps minor did not differ significantly between preterm and full term groups. There are several possible interpretations for the lack of differences within these periventricular tracts. One possibility is that injuries in these pathways observed in other studies at term equivalent ages [6, 44, 47] may have resolved by the ages studied here. Age-related increases in FA are well documented in children [88–90] and could thus compensate for initial decreases in FA. A second possibility is that the differences resolved because the present sample of preterm children received enhanced education and therapy. White matter microstructural changes are demonstrated to occur in response to a range of environmental experiences in numerous animal species [91] and in response to intensive behavioral remediation in humans [92–96]. Early injury to the forceps minor and corticospinal tracts [6, 44, 47] could thus recover as children practice the visual or motor skills dependent on axonal projections that comprise these tracts.

The lack of significant group differences within corticospinal tracts and the forceps minor may also be explained by several methodological differences across studies. The procedures we used for identifying and segmenting tracts were designed to characterize core tract regions. Our procedures purposely excluded peripheral tract segments that are more variable and susceptible to partial voluming [42]. The present methods may thus be insensitive to group differences, that in previous voxel-based studies, have been shown to occur in regions of the corticospinal tract that extend beyond the core tract regions analyzed here [6, 38]. By ignoring peripheral tract regions, the present methods may also be insensitive to group differences that may occur in more distal regions that can otherwise be captured by mean tract measures generated from averaging diffusion properties over the entire length of white matter pathways [10]. Alternatively, it is possible that by isolating only core tract regions, the present methods capture fibers that may be robust to injury and dysmaturation or have enhanced capabilities for recovery. Finally, it is also possible that the present pattern of observations relates to specific clinical characteristics of the present sample. Investigating these possibilities will ultimately require replication in other samples, as well as longitudinal analyses to understand whether FA differences observed at younger ages change in association with age or specific environmental experiences.

### Group differences in non-periventricular tracts

In the present study, we observed evidence for microstructural differences between the preterm and full term groups in non-periventricular tracts, including the anterior thalamic radiations and the uncinate fasciculus. These findings are consistent with previous tractography studies that have found group FA differences within the left and right anterior thalamic radiations in preterm neonates with varying degrees of white matter abnormalities [97], as well as findings from a tractography study in which a group of preterm children demonstrated reduced FA in both the left and right uncinate compared to the full term group [9]. Together with these findings, the present pattern of results suggests that white matter changes associated with preterm birth are also diffuse [4, 16].

Evidence for microstructural differences within the anterior thalamic radiations and uncinate fasciculus also have important implications for understanding the range of neurodevelopmental outcomes experienced by children born preterm. Axonal projections contained within the anterior thalamic radiations are known to connect the thalamus, striatum, and frontal lobes [98]. The uncinate fasciculus connects regions of the anterior temporal and inferior frontal lobes and has been implicated in higher-order cognitive and linguistic skills [99]. Evidence for injury or dysmaturity to fiber connections of the anterior thalamic radiations in preterm children may account, in part, for individual variations in behavioral abilities thought to involve functions of the frontal lobes, including attention [100–102] and working memory processes [103–105]. Similarly, evidence for injury or dysmaturity within the uncinate fasciculus may account for the range of language and reading outcomes observed in preterm children [106–109].

The present study cannot determine whether the group differences observed in the anterior thalamic radiations or uncinate fasciculus reflect the long-term consequences of early injury or altered maturation, or differences related to developmental experiences after birth. Multiple sources of evidence indicate that myelination of supramodal frontal and temporal cortices has a protracted developmental time-course relative to unimodal cortices responsible for supporting primary sensorimotor processes [110, 111]. Longitudinal studies will be important for determining whether the observed group differences seen here and in other studies of older preterm children and adults [9–12] reflect sustained injuries from birth or delayed differences reflective of altered maturation in the myelination of these pathways or compensatory processes. In addition, intervention studies will be important for determining whether microstructural properties of white matter tracts are capable of changing in association with specific educational or medical therapies for improving functional outcomes. Such findings would have important implications for understanding neural basis of plasticity following early white matter injury and functional characteristics of white matter pathways in general.

### Gestational Age

In this study we observed very few statistically significant associations between FA and gestational age at birth of the preterm participants. Studies find that behavioral outcomes of children born preterm tend to correlate with gestational age at birth, including cerebral palsy, which itself is associated with brain injury [112]. Studies have also found that selected brain findings, such as bi-parietal width, are also correlated with gestational age at birth [113]. Such relationships suggest that white matter properties associated with poor outcomes might also be associated with gestational age. Indeed, studies have observed associations between white matter properties in relation to gestational age at birth [45, 49, 114–119]. However, it has also been shown that accumulated medical variables over the course of the neonatal hospitalization are more predictive than gestational age [120, 121]. Several additional explanations may account for the minimal significant correlations observed here. First, sample sizes must be adequately powered to observe associations. The present sample size may be too small to detect such correlations over and above individual differences within the preterm group. Second, correlations between diffusion properties and gestational age may be strongest at the limits of viability, similar to correlations with behavioral outcomes [122]. Here, the gestational age range of the participants in this study was broad. Third, white matter injuries may be associated with the timing of adverse complications from preterm birth, such as hypoxia-ischemia and infection, and these events may occur pre-, peri- or post- natally. Diffusion properties may therefore be more closely associated with the presence of acute adverse medical events than with gestational age per se [47]. Samples larger than the present one would be required to investigate such associations and will be useful for performing sub-group analyses to determine whether group differences on the basis of preterm birth may be driven by specific gestational age-ranges.

### Variations in the diffusion properties across tracts

Across the different tracts assessed in this study, we found that the preterm group had both decreased and increased FA relative to the full term group. Such findings suggest that the underlying neurobiological factors that contribute to FA differences vary across and along the trajectory of individual white matter tracts.

Evidence for decreased FA within anterior segments of the right inferior fronto-occipital fasciculus and bilateral uncinate fasciculus in the preterm group is consistent with several previous studies of preterm children and adults at various ages [9–12]. Interestingly, decreased FA was not detected within this same sample using a different diffusion tensor imaging analytic strategy [38]. The different results using the two methods suggest greater sensitivity of tractography to group differences in this clinical group. The primary assumption about decreased FA in children born preterm is that the results suggest decreased myelination. Groeschel and colleagues (2014) specifically postulated that decreased FA in the preterm group would occur in segments of the corpus callosum and internal capsule where there was a single fiber orientation. In these regions, increased radial diffusivity appeared to be the largest contributor to decreased FA [11]. Our results were quite similar for the anterior segments of the right inferior fronto-occipital fasciculus where we too found decreased FA and increased radial diffusivity. However, within the uncinate fasciculus bilaterally and also within anterior segments of the right inferior fronto-occipital fasciculus, the preterm group in the current study had decreased FA and elevated AD. Any explanations for decreased FA in these tracts that we might offer would be speculative. Nonetheless, taken together, these variations in diffusion properties suggest that underlying tissue properties related to myelin and axonal status may vary and interact in different tracts with both changes leading to decreased FA.

Increased FA in several tracts of the preterm group is consistent with findings from this cohort using a different dMRI analytic strategy [38], as well as other dMRI studies of children, adolescents and adults born preterm [11, 37, 39, 40]. Groeschel et al., (2014) located increased FA to predominantly crossing-fiber regions where the interpretation of radial and axial diffusivity is not meaningful. They attributed increased FA to a decrease in anisotropy of a contributing fiber bundle. A similar explanation would be a reduction in the number or density of crossing fibers from axonal loss in the preterm group that could result in increased FA [28]. Alternatively, increased FA may reflect increased myelination from compensatory processes. However, such interpretations for FA are only likely to reflect changes in myelin status within white matter tracts demonstrating single fiber orientations [22]. Studies have estimated that the majority of white matter voxels (60–90%) contain crossing fibers [29]. Taken together, such findings suggest that the observed patterns for both increased and decreased FA in the present study may reflect inter- and intra- tract variations in the proportions of crossing fibers. Distinguishing amongst these interpretations for FA is likely to require additional neuroimaging techniques for obtaining voxel-based estimates for the proportion of crossing fibers [123, 124] and tissue myelination [125, 126]. In general, combining such methods with FA measurements is likely to afford greater understandings for the neurobiological underpinnings of both decreased and increased FA observed in preterm children at different ages. For example, this combination may help to clarify whether increased FA within specific pathways is associated with high myelin content, or alternatively whether increased FA in the context of low myelin content may reflect decreased numbers of crossing fibers possibly related to axonal loss.

## Limitations

The current group of children born preterm represented a convenience sample with a wide age range. Given the large age range of the current sample, it is possible that developmental changes in FA could have accounted for the present group differences. However, we think that this possibility was low because we did not observe significant main effects of age for any white matter tract in omnibus tests performed to identify group differences. Replication studies within specific age groups and/or larger sample sizes will be important for establishing the generalizability of the present findings.

## Conclusions and Future Directions

This dMRI tractography study confirms that differences in white matter microstructure observed for children born preterm persist into adolescence. Group differences are found in both periventricular and non-periventricular white matter tracts. The pattern of results is complex: the preterm group shows both increased and decreased FA values in different segments of the identified white matter tracts compared to the full term group. Future studies using quantitative MRI measurements are needed to understand the sources of the identified differences. In addition, longitudinal analyses may help to determine how patterns of prematurity-related white matter differences change from birth to adulthood. Future studies involving larger samples of preterm and full term children than the current study will be important for understanding how other factors that are known to affect white matter development, such as sex and birth weight [77, 127, 128], interact with group differences. Finally, future studies should consider the functional implications of the structural differences identified here between children born preterm and full term.

## Supporting Information

S1 Fig Spearman correlation between GA and mean FA from the right IFOF where significant group differences were observed (location 18–19). (DOCX)

S1 Table Fiber Tract Identification in Full Term and Preterm Subjects. (DOCX)

S2 Table Spearman Correlations between mean FA of 18 Cerebral White Matter Tracts and Age at Diffusion Imaging for Preterm and Full Term Groups. (DOCX)

S3 Table Spearman Correlations between mean MD of 18 Cerebral White Matter Tracts and Age at Diffusion Imaging for Preterm and Full Term Groups. (DOCX)

S4 Table Group comparison between mean tract volumes of 18 cerebral white matter tracts and whole brain fiber group. (DOCX)

S5 Table ANCOVA results for the comparison of preterm and full term MD profiles. (DOCX)

S1 Text Translational Head Motion. (DOCX)

## References

[1] Martin JA, Hamilton BE, Ventura SJ, Osterman MJ, Mathews TJ. Births: final data for 2011. Natl Vital Stat Rep. 2013;62(1):1–69. . 24974591

[2] Aylward GP. Neurodevelopmental outcomes of infants born prematurely. J Dev Behav Pediatr. 2014;35(6):394–407. 10.1097/01.DBP.0000452240.39511.d4 . 25007063

[3] Back SA. Perinatal white matter injury: the changing spectrum of pathology and emerging insights into pathogenetic mechanisms. Mental Retard Dev D R. 2006;12(2):129–40. 10.1002/mrdd.20107 16807910

[4] Back SA, Riddle A, McClure MM. Maturation-dependent vulnerability of perinatal white matter in premature birth. Stroke. 2007;38(2 Suppl):724–30. 10.1161/01.STR.0000254729.27386.05 . 17261726

[5] Volpe JJ. Brain injury in premature infants: a complex amalgam of destructive and developmental disturbances. Lancet Neurol. 2009;8(1):110–24. 10.1016/S1474-4422(08)70294-1 19081519 PMC2707149

[6] Anjari M, Srinivasan L, Allsop JM, Hajnal JV, Rutherford MA, Edwards AD, et al Diffusion tensor imaging with tract-based spatial statistics reveals local white matter abnormalities in preterm infants. Neuroimage. 2007;35(3):1021–7. 17344066 10.1016/j.neuroimage.2007.01.035

[7] Pannek K, Scheck SM, Colditz PB, Boyd RN, Rose SE. Magnetic resonance diffusion tractography of the preterm infant brain: a systematic review. Dev Med Child Neurol. 2014;56(2):113–24. Epub 2013/10/10. 10.1111/dmcn.12250 . 24102176

[8] Arzoumanian Y, Mirmiran M, Barnes PD, Woolley K, Ariagno RL, Moseley ME, et al Diffusion tensor brain imaging findings at term-equivalent age may predict neurologic abnormalities in low birth weight preterm infants. AJNR. 2003;24(8):1646–53. . 13679287 PMC7974006

[9] Constable RT, Ment LR, Vohr BR, Kesler SR, Fulbright RK, Lacadie C, et al Prematurely born children demonstrate white matter microstructural differences at 12 years of age, relative to term control subjects: an investigation of group and gender effects. Pediatrics. 2008;121(2):306–16. 10.1542/peds.2007-0414 18245422

[10] de Kieviet JF, Pouwels PJ, Lafeber HN, Vermeulen RJ, van Elburg RM, Oosterlaan J. A crucial role of altered fractional anisotropy in motor problems of very preterm children. Euro J Paediatr Neuro. 2014;18(2):126–33. 10.1016/j.ejpn.2013.09.004 24119780

[11] Groeschel S, Tournier JD, Northam GB, Baldeweg T, Wyatt J, Vollmer B, et al Identification and interpretation of microstructural abnormalities in motor pathways in adolescents born preterm. Neuroimage. 2014;87:209–19. Epub 2013/11/05. 10.1016/j.neuroimage.2013.10.034S1053-8119(13)01057-4 [pii]. . 24185027

[12] Jo HM, Cho HK, Jang SH, Yeo SS, Lee E, Kim HS, et al A comparison of microstructural maturational changes of the corpus callosum in preterm and full-term children: a diffusion tensor imaging study. Neuroradiology. 2012;54(9):997–1005. 10.1007/s00234-012-1042-8 22562691

[13] Kontis D, Catani M, Cuddy M, Walshe M, Nosarti C, Jones D, et al Diffusion tensor MRI of the corpus callosum and cognitive function in adults born preterm. Neuroreport. 2009;20(4):424–8. Epub 2009/02/17. 10.1097/WNR.0b013e328325a8f9 . 19218872

[14] Gano D, Andersen SK, Partridge JC, Bonifacio SL, Xu D, Glidden DV, et al Diminished white matter injury over time in a cohort of premature newborns. J Pediatr. 2015;166(1):39–43. 10.1016/j.jpeds.2014.09.009 25311709 PMC4274204

[15] Back SA, Luo NL, Borenstein NS, Levine JM, Volpe JJ, Kinney HC. Late oligodendrocyte progenitors coincide with the developmental window of vulnerability for human perinatal white matter injury. J Neurosci. 2001;21(4):1302–12. Epub 2001/02/13. doi: 21/4/1302 [pii]. . 11160401 10.1523/JNEUROSCI.21-04-01302.2001 PMC6762224

[16] Back SA, Rosenberg PA. Pathophysiology of glia in perinatal white matter injury. Glia. 2014;62:1790–815. Epub 2014/04/02. 10.1002/glia.22658 . 24687630 PMC4163108

[17] Back SA, Miller SP. Brain injury in premature neonates: a primary cerebral dysmaturation disorder? Ann Neurol. 2014;75(4):469–86. Epub 2014/03/13. 10.1002/ana.24132 . 24615937 PMC5989572

[18] Riddle A, Luo N, Manese M, Beardsley D, Green L, Rorvik D, et al Spatial heterogeneity in oligodendrocyte lineage maturation and not cerebral blood flow predicts fetal ovine periventricular white matter injury. J Neurosci. 2006;26(11):3045–55. 16540583 10.1523/JNEUROSCI.5200-05.2006 PMC6673975

[19] Basser PJ, Pierpaoli C. Microstructural and physiological features of tissues elucidated by quantitative-diffusion-tensor MRI. J Magn Reson. 1996;111(3):209–19. . 8661285 10.1006/jmrb.1996.0086

[20] Feldman HM, Yeatman JD, Lee ES, Barde LHF, Gaman-Bean S. Diffusion tensor imaging: a review for pediatric researchers and clinicians. J Dev Behav Pediatr. 2010;31(4):346–56. 10.1097/DBP.0b013e3181dcaa8b . 20453582 PMC4245082

[21] Beaulieu C. The basis of anisotropic water diffusion in the nervous system—a technical review. NMR in Biomedicine. 2002;15(7–8):435–55. 10.1002/nbm.782 12489094

[22] De Santis S, Drakesmith M, Bells S, Assaf Y, Jones DK. Why diffusion tensor MRI does well only some of the time: Variance and covariance of white matter tissue microstructure attributes in the living human brain. Neuroimage. 2014;89(0):35–44. 10.1016/j.neuroimage.2013.12.003 24342225 PMC3988851

[23] Kumar R, Macey PM, Woo MA, Alger JR, Harper RM. Diffusion tensor imaging demonstrates brainstem and cerebellar abnormalities in congenital central hypoventilation syndrome. Pediatr Res. 2008;64(3):275–80. 10.1203/PDR.0b013e31817da10a 18458651 PMC2682538

[24] Kumar R, Macey PM, Woo MA, Harper RM. Rostral brain axonal injury in congenital central hypoventilation syndrome. J Neurosci Res. 2010;88(10):2146–54. 10.1002/jnr.22385 . 20209631

[25] Kumar R, Nguyen HD, Macey PM, Woo MA, Harper RM. Regional brain axial and radial diffusivity changes during development. J Neurosci Res. 2012;90(2):346–55. 10.1002/jnr.22757 21938736 PMC3237749

[26] Song SK, Yoshino J, Le TQ, Lin SJ, Sun SW, Cross AH, et al Demyelination increases radial diffusivity in corpus callosum of mouse brain. Neuroimage. 2005;26(1):132–40. 10.1016/j.neuroimage.2005.01.028 . 15862213

[27] Song S-K, Sun S-W, Ramsbottom MJ, Chang C, Russell J, Cross AH. Dysmyelination revealed through MRI as increased radial (but unchanged axial) diffusion of water. Neuroimage. 2002;17(3):1429–36. 12414282 10.1006/nimg.2002.1267

[28] Jones D. Challenges and limitations of quantifying brain connectivity in vivo with diffusion MRI. Imaging Medicine. 2010;2(3):341–55.

[29] Jeurissen B, Leemans A, Tournier JD, Jones DK, Sijbers J. Investigating the prevalence of complex fiber configurations in white matter tissue with diffusion magnetic resonance imaging. Hum Brain Mapp. 2013;34(11):2747–66. Epub 2012/05/23. 10.1002/hbm.22099 . 22611035 PMC6870534

[30] Gimenez M, Miranda MJ, Born AP, Nagy Z, Rostrup E, Jernigan TL. Accelerated cerebral white matter development in preterm infants: a voxel-based morphometry study with diffusion tensor MR imaging. Neuroimage. 2008;41(3):728–34. 10.1016/j.neuroimage.2008.02.029 18430590

[31] Rose SE, Hatzigeorgiou X, Strudwick MW, Durbridge G, Davies PSW, Colditz PB. Altered white matter diffusion anisotropy in normal and preterm infants at term-equivalent age. Magn Reson Med. 2008;60(4):761–7. 10.1002/mrm.21689 18816850

[32] Andrews JS, Ben-Shachar M, Yeatman JD, Flom LL, Luna B, Feldman HM. Reading performance correlates with white-matter properties in preterm and term children. Dev Med Child Neurol. 2010;52(6):e94–100. Epub 2009/09/15. 10.1111/j.1469-8749.2009.03456.x DMCN3456 [pii]. 19747208 PMC2892255

[33] Nagy Z, Westerberg H, Skare S, Andersson J, LIlja A, Flodmark O, et al Preterm children have disturbances of white matter at 11 years of age as shown by diffusion tensor imaging. Pediatr Res. 2003;54:672–9. 12904607 10.1203/01.PDR.0000084083.71422.16

[34] Yung A, Poon G, Qiu D-Q, Chu J, Lam B, Leung C, et al White matter volume and anisotropy in preterm children: a pilot study of neurocognitive correlates. Pediatr Res. 2007;61(6):732–6. 17426647 10.1203/pdr.0b013e31805365db

[35] Mullen KM, Vohr BR, Katz KH, Schneider KC, Lacadie C, Hampson M, et al Preterm birth results in alterations in neural connectivity at age 16 years. Neuroimage. 2011;54(4):2563–70. 10.1016/j.neuroimage.2010.11.019 21073965 PMC3020252

[36] Skranes J, Vangberg TR, Kulseng S, Indredavik MS, Evensen KAI, Martinussen M, et al Clinical findings and white matter abnormalities seen on diffusion tensor imaging in adolescents with very low birth weight. Brain. 2007;130(Pt 3):654–66. 10.1093/brain/awm001 . 17347255

[37] Vangberg TR, Skranes J, Dale AM, Martinussen M, Brubakk AM, Haraldseth O, et al Changes in white matter diffusion anisotropy in adolescents born prematurely. Neuroimage. 2006;32(4):1538–48. . 16843682 10.1016/j.neuroimage.2006.04.230

[38] Feldman HM, Lee ES, Loe IM, Yeom KW, Grill-Spector K, Luna B. White matter microstructure on diffusion tensor imaging is associated with conventional magnetic resonance imaging findings and cognitive function in adolescents born preterm. Dev Med Child Neurol. 2012;54(9):809–14. 10.1111/j.1469-8749.2012.04378.x . 22803787 PMC3683593

[39] Allin MPG, Kontis D, Walshe M, Wyatt J, Barker GJ, Kanaan RAA, et al White matter and cognition in adults who were born preterm. PLoS ONE. 2011;6(10):e24525 10.1371/journal.pone.0024525 22022357 PMC3192037

[40] Eikenes L, Løhaugen GC, Brubakk A-M, Skranes J, Håberg AK. Young adults born preterm with very low birth weight demonstrate widespread white matter alterations on brain DTI. Neuroimage. 2011;54(3):1774–85. 10.1016/j.neuroimage.2010.10.037 20965255

[41] Frye R, Hasan K, Malmberg B, Desouza L, Smith K, Landry S. Superior longitudinal fasciculus and cognitive dysfunction in adolescents born preterm and at term. Dev Med Child Neurol. 2010;52:760–6. 10.1111/j.1469-8749.2010.03633.x 20187879 PMC2910222

[42] Yeatman JD, Dougherty RF, Myall NJ, Wandell BA, Feldman HM. Tract profiles of white matter properties: automating fiber-tract quantification. PLoS ONE. 2012;7(11):e49790 10.1371/journal.pone.0049790 23166771 PMC3498174

[43] Yeatman JD, Dougherty RF, Rykhlevskaia E, Sherbondy AJ, Deutsch GK, Wandell BA, et al Anatomical properties of the arcuate fasciculus predict phonological and reading skills in children. J Cog Neuro. 2011;23(11):3304–17. 10.1162/jocn_a_00061 PubMed PMID: 3970416825482263677related:fbwaDRHBGTcJ. PMC3214008 21568636

[44] Thompson DK, Inder T, Faggian N, Johnston L, Warfield SK, Anderson PJ, et al Characterization of the corpus callosum in very preterm and full term infants utilizing MRI. Neuroimage. 2011;55:479–90. 10.1016/j.neuroimage.2010.12.025 21168519 PMC3035727

[45] Ball G, Boardman JP, Aljabar P, Pandit A, Arichi T, Merchant N, et al The influence of preterm birth on the developing thalamocortical connectome. Cortex. 2013;49(6):1711–21. 10.1016/j.cortex.2012.07.006 . 22959979

[46] Kaur S, Powell S, He L, Pierson CR, Parikh NA. Reliability and repeatability of quantitative tractography methods for mapping structural white matter connectivity in preterm and term infants at term-equivalent age. PLoS One. 2014;9(1):e85807 10.1371/journal.pone.0085807 24475054 PMC3901659

[47] Adams E, Chau V, Poskitt KJ, Grunau RE, Synnes A, Miller SP. Tractography-based quantitation of corticospinal tract development in premature newborns. J Pediatr. 2010;156(6):882–8. 10.1016/j.jpeds.2009.12.030 20227731 PMC3760842

[48] Liu Y, Balériaux D, Kavec M, Metens T, Absil J, Denolin V, et al Structural asymmetries in motor and language networks in a population of healthy preterm neonates at term equivalent age: a diffusion tensor imaging and probabilistic tractography study. Neuroimage. 2010;51(2):783–8. 10.1016/j.neuroimage.2010.02.066 20206706

[49] Hasegawa T, Yamada K, Morimoto M, Morioka S, Tozawa T, Isoda K, et al Development of corpus callosum in preterm infants is affected by the prematurity: in vivo assessment of diffusion tensor imaging at term-equivalent age. Pediatr Res. 2011;69(3):249–54. 10.1203/PDR.0b013e3182084e54 21131895

[50] Melbourne A, Kendall GS, Cardoso MJ, Gunny R, Robertson NJ, Marlow N, et al Preterm birth affects the developmental synergy between cortical folding and cortical connectivity observed on multimodal MRI. Neuroimage. 2014;89:23–34. 10.1016/j.neuroimage.2013.11.048 24315841

[51] Berman JI, Mukherjee P, Partridge SC, Miller SP, Ferriero DM, Barkovich AJ, et al Quantitative diffusion tensor MRI fiber tractography of sensorimotor white matter development in premature infants. Neuroimage. 2005;27(4):862–71. 15978841 10.1016/j.neuroimage.2005.05.018

[52] Bassi L, Ricci D, Volzone A, Allsop JM, Srinivasan L, Pai A, et al Probabilistic diffusion tractography of the optic radiations and visual function in preterm infants at term equivalent age. Brain. 2008;131(2):573–82. 18222994 10.1093/brain/awm327

[53] Guevara P, Duclap D, Poupon C, Marrakchi-Kacem L, Fillard P, Le Bihan D, et al Automatic fiber bundle segmentation in massive tractography datasets using a multi-subject bundle atlas. Neuroimage. 2012;61(4):1083–99. 10.1016/j.neuroimage.2012.02.071 . 22414992

[54] Gong G, Jiang T, Zhu C, Zang Y, Wang F, Xie S, et al Asymmetry analysis of cingulum based on scale-invariant parameterization by diffusion tensor imaging. Hum Brain Mapp. 2005;24(2):92–8. 10.1002/hbm.20072 . 15455461 PMC6871701

[55] Myall NJ, Yeom KW, Yeatman JD, Gaman-Bean S, Feldman HM. Case series: fractional anisotropy along the trajectory of selected white matter tracts in adolescents born preterm with ventricular dilation. J Child Neurol. 2013;28(6):774–80. 10.1177/0883073812449693 22859695 PMC4277874

[56] Mori S, Wakana S, Van Zijl PC, Nagae-Poetscher L. MRI atlas of human white matter. 2005. 10.1148/radiol.2301021640 14645885

[57] Lee ES, Yeatman JD, Luna B, Feldman HM. Specific language and reading skills in school-aged children and adolescents are associated with prematurity after controlling for IQ. Neuropsychologia. 2011;49(5):906–13. 10.1016/j.neuropsychologia.2010.12.038 21195100 PMC3078177

[58] Loe IM, Lee ES, Luna B, Feldman HM. Executive function skills are associated with reading and parent-rated child function in children born prematurely. Early Hum Dev. 2012;88(2):111–8. 10.1016/j.earlhumdev.2011.07.018 21849240 PMC3660611

[59] Loe IM, Lee ES, Luna B, Feldman HM. Behavior problems of 9–16year old preterm children: biological, sociodemographic, and intellectual contributions. Early Hum Dev. 2011;87(4):247–52. 10.1016/j.earlhumdev.2011.01.023 21316875 PMC3180905

[60] Loe IM, Luna B, Bledsoe IO, Yeom KW, Fritz BL, Feldman HM. Oculomotor assessments of executive function in preterm children. J Pediatr. 2012;161(3):427–33. 10.1016/j.jpeds.2012.02.037 22480696 PMC3638733

[61] Feldman HM, Lee ES, Yeatman JD, Yeom KW. Language and reading skills in school-aged children and adolescents born preterm are associated with white matter properties on diffusion tensor imaging. Neuropsychologia. 2012;50:3348–62. 10.1016/j.neuropsychologia.2012.10.014 23088817 PMC3631607

[62] Yeatman JD, Feldman HM. Neural plasticity after pre-linguistic injury to the arcuate and superior longitudinal fasciculi. Cortex. 2013;49(1):301–11. 10.1016/j.cortex.2011.08.006 21937035 PMC3259257

[63] Wechsler D. Wechsler Abbreviated Scale of Intelligence. San Antonio, TX: The Pscyhological Corporation/ A brand of Harcourt Assessment, Inc; 1999.

[64] Oldfield RC. The assessment and analysis of handedness: the Edinburgh inventory. Neuropsychologia. 1971;9(1):97–113. . 5146491 10.1016/0028-3932(71)90067-4

[65] Inder TE, Wells SJ, Mogridge NB, Spencer C, Volpe JJ. Defining the nature of the cerebral abnormalities in the premature infant: a qualitative magnetic resonance imaging study. J Pediatr. 2003;143(2):171–9. 10.1067/S0022-3476(03)00357-3 . 12970628

[66] Inder T, Huppi PS, Zientara GP, Maier SE, Jolesz FA, di Salvo D, et al Early detection of periventricular leukomalacia by diffusion-weighted magnetic resonance imaging techniques. J Pediatr. 1999;134(5):631–4. . 10228300 10.1016/s0022-3476(99)70251-9

[67] Northam GB, Liegeois F, Chong WK, Wyatt JS, Baldeweg T. Total brain white matter is a major determinant of IQ in adolescents born preterm. Ann Neurol. 2011;69(4):702–11. 10.1002/ana.22263 . 21391229

[68] Rohde GK, Barnett AS, Basser PJ, Marenco S, Pierpaoli C. Comprehensive approach for correction of motion and distortion in diffusion-weighted MRI. Magn Reson Med. 2004;51(1):103–14. . 14705050 10.1002/mrm.10677

[69] Chang LC, Jones DK, Pierpaoli C. RESTORE: robust estimation of tensors by outlier rejection. Magn Reson Med. 2005;53(5):1088–95. . 15844157 10.1002/mrm.20426

[70] Conturo TE, Lori NF, Cull TS, Akbudak E, Snyder AZ, Shimony JS, et al Tracking neuronal fiber pathways in the living human brain. Proc Natl Acad Sci U S A. 1999;96(18):10422–7. 10468624 10.1073/pnas.96.18.10422 PMC17904

[71] Mori S, Crain BJ, Chacko V, Van Zijl P. Three‐dimensional tracking of axonal projections in the brain by magnetic resonance imaging. Ann Neurol. 1999;45(2):265–9. 9989633 10.1002/1531-8249(199902)45:2<265::aid-ana21>3.0.co;2-3

[72] Press W, Teukolsky S, Vetterling W, Flannery B. Numerical Recipes in C++:The Art of Scientific Computing. Cambridge, UK: Cambridge Univ Press 2002.

[73] Wakana S, Caprihan A, Panzenboeck MM, Fallon JH, Perry M, Gollub RL, et al Reproducibility of quantitative tractography methods applied to cerebral white matter. Neuroimage. 2007;36(3):630–44. 10.1016/j.neuroimage.2007.02.049 17481925 PMC2350213

[74] Friston KJ, Ashburner J. Generative and recognition models for neuroanatomy. Neuroimage. 2004;23(1):21–4. 10.1016/j.neuroimage.2004.04.021 . 15325348

[75] Kronfeld-Duenias V, Amir O, Ezrati-Vinacour R, Civier O, Ben-Shachar M. The frontal aslant tract underlies speech fluency in persistent developmental stuttering. Brain Struct Funct. 2014 10.1007/s00429-014-0912-8 . 25344925

[76] Lebel C, Beaulieu C. Longitudinal development of human brain wiring continues from childhood into adulthood. J Neurosci. 2011;31(30):10937–47. 10.1523/JNEUROSCI.5302-10.2011 . 21795544 PMC6623097

[77] Johnson RT, Yeatman JD, Wandell BA, Buonocore MH, Amaral DG, Nordahl CW. Diffusion properties of major white matter tracts in young, typically developing children. Neuroimage. 2013;88:143–54. 24269274 10.1016/j.neuroimage.2013.11.025 PMC4029877

[78] Mauchly JW. Significance test for sphericity of a normal n-variate distribution. The Annals of Mathematical Statistics. 1940;11(2):204–9.

[79] Benjamini Y, Hochberg Y. Controlling the false discovery rate: A practical and powerful approach to multiple testing. J Royal Stat Soc B Met. 1995;57(Series B):289–300.

[80] Nichols TE, Holmes AP. Nonparametric permutation tests for functional neuroimaging: a primer with examples. Hum Brain Mapp. 2002;15(1):1–25. Epub 2001/12/18. 10.1002/hbm.1058 [pii]. . 11747097 PMC6871862

[81] Catani M, Allin MP, Husain M, Pugliese L, Mesulam MM, Murray RM, et al Symmetries in human brain language pathways correlate with verbal recall. Proc Natl Acad Sci U S A. 2007;104(43):17163–8. 17939998 10.1073/pnas.0702116104 PMC2040413

[82] Lebel C, Beaulieu C. Lateralization of the arcuate fasciculus from childhood to adulthood and its relation to cognitive abilities in children. Hum Brain Mapp. 2009;30(11):3563–73. 10.1002/hbm.20779 19365801 PMC6870654

[83] Mishra A, Anderson AW, Wu X, Gore JC, Ding Z. An improved Bayesian tensor regularization and sampling algorithm to track neuronal fiber pathways in the language circuit. Med Phys. 2010;37(8):4274–87. 20879588 10.1118/1.3456113 PMC2921424

[84] Volpe JJ. Brain injury in the premature infant: overview of clinical aspects, neuropathology, and pathogenesis. Semin Pediatr Neurol. 1998;5(3):135–51. Epub 1998/10/20. . 9777673 10.1016/s1071-9091(98)80030-2

[85] Volpe JJ. Intraventricular hemorrhage and brain injury in the premature infant. Neuropathology and pathogenesis. Clin Perinatol. 1989;16(2):361–86. Epub 1989/06/01. . 2663307

[86] Elovitz MA, Mrinalini C. Animal models of preterm birth. Trends Endocrin Met. 2004;15(10):479–87. 10.1016/j.tem.2004.10.009 . 15541647

[87] Wisnowski JL, Schmithorst VJ, Rosser T, Paquette L, Nelson MD, Haynes RL, et al Magnetic resonance spectroscopy markers of axons and astrogliosis in relation to specific features of white matter injury in preterm infants. Neuroradiology. 2014;56(9):771–9. 10.1007/s00234-014-1380-9 . 24903580 PMC9242581

[88] Asato MR, Terwilliger R, Woo J, Luna B. White matter development in adolescence: a DTI study. Cereb Cortex. 2010;20(9):2122–31. 10.1093/cercor/bhp282 20051363 PMC2923214

[89] Lebel C, Gee M, Camicioli R, Wieler M, Martin W, Beaulieu C. Diffusion tensor imaging of white matter tract evolution over the lifespan. Neuroimage. 2012;60(1):340–52. 10.1016/j.neuroimage.2011.11.094 22178809

[90] Yeatman JD, Dougherty RF, Myall NJ, Wandell BA, Feldman HM. Tract profiles of white matter properties: automating fiber-tract quantification. PLoS ONE [Electronic Resource]. 2012;7(11):e49790 10.1371/journal.pone.0049790 23166771 PMC3498174

[91] Fields RD. White matter in learning, cognition and psychiatric disorders. Trends Neurosci. 2008;31(7):361–70. Epub 2008/06/10. 10.1016/j.tins.2008.04.001 S0166-2236(08)00132-X [pii]. 18538868 PMC2486416

[92] Scholz J, Klein MC, Behrens TE, Johansen-Berg H, Scholz J, Klein MC, et al Training induces changes in white-matter architecture. Nat Neurosci. 2009;12(11):1370–1. 10.1038/nn.2412 19820707 PMC2770457

[93] Keller TA, Just MA. Altering cortical connectivity: remediation-induced changes in the white matter of poor readers. Neuron. 2009;64(5):624–31. 10.1016/j.neuron.2009.10.018 20005820 PMC2796260

[94] Als H, Duffy FH, McAnulty GB, Rivkin MJ, Vajapeyam S, Mulkern RV, et al Early experience alters brain function and structure. Pediatrics. 2004;113(4):846–57. Epub 2004/04/03. . 15060237 10.1542/peds.113.4.846

[95] Sagi Y, Tavor I, Hofstetter S, Tzur-Moryosef S, Blumenfeld-Katzir T, Assaf Y. Learning in the fast lane new insights into neuroplasticity. Neuron. 2012;73:1195–203. 10.1016/j.neuron.2012.01.025 22445346

[96] Hoeft F, McCandliss BD, Black JM, Gantman A, Zakerani N, Hulme C, et al Neural systems predicting long-term outcome in dyslexia. Proc Natl Acad Sci USA. 2011;108(1):361–6. 10.1073/pnas.1008950108 21173250 PMC3017159

[97] Liu Y, Aeby A, Baleriaux D, David P, Absil J, De Maertelaer V, et al White matter abnormalities are related to microstructural changes in preterm neonates at term-equivalent age: a diffusion tensor imaging and probabilistic tractography study. Am J Neuroradiol. 2012;33(5):839–45. 10.3174/ajnr.A2872 . 22241389 PMC7968822

[98] Coenen VA, Panksepp J, Hurwitz TA, Urbach H, Madler B. Human medial forebrain bundle (MFB) and anterior thalamic radiation (ATR): imaging of two major subcortical pathways and the dynamic balance of opposite affects in understanding depression. J Neuropsychiatry Clin Neurosci. 2012;24(2):223–36. 10.1176/appi.neuropsych.11080180 . 22772671

[99] Von Der Heide RJ, Skipper LM, Klobusicky E, Olson IR. Dissecting the uncinate fasciculus: disorders, controversies and a hypothesis. Brain. 2013;136(6):1692–707. 10.1093/brain/awt094 23649697 PMC3673595

[100] Skranes J, Vangberg TR, Kulseng S, Indredavik MS, Evensen KAI, Martinussen M, et al Clinical findings and white matter abnormalities seen on diffusion tensor imaging in adolescents with very low birth weight. Brain. 2007;130(3):654–66. 10.1093/brain/awm001 17347255

[101] Johnson S, Marlow N. Preterm birth and childhood psychiatric disorders. Pediatr Res. 2011;69(5 Pt 2):11R–8R. 10.1203/PDR.0b013e318212faa0 21289534

[102] Hack M, Youngstrom EA, Cartar L, Schluchter M, Taylor HG, Flannery D, et al Behavioral outcomes and evidence of psychopathology among very low birth weight infants at age 20 years. Pediatrics. 2004;114(4):932–40. . 15466087 10.1542/peds.2003-1017-L

[103] Aarnoudse-Moens CS, Weisglas-Kuperus N, van Goudoever JB, Oosterlaan J. Meta-analysis of neurobehavioral outcomes in very preterm and/or very low birth weight children. Pediatrics. 2009;124(2):717–28. 10.1542/peds.2008-2816 19651588

[104] Anderson PJ, Doyle LW, Victorian Infant Collaborative Study G. Executive functioning in school-aged children who were born very preterm or with extremely low birth weight in the 1990s. Pediatrics. 2004;114(1):50–7. . 15231907 10.1542/peds.114.1.50

[105] Skranes J, Lohaugen GC, Martinussen M, Indredavik MS, Dale AM, Haraldseth O, et al White matter abnormalities and executive function in children with very low birth weight. Neuroreport. 2009;20(3):263–6. . 19444947 10.1097/wnr.0b013e32832027fe

[106] Bhutta AT, Cleves MA, Casey PH, Cradock MM, Anand KJS. Cognitive and behavioral outcomes of school-aged children who were born preterm: a meta-analysis. JAMA. 2002;288(6):728–37. 12169077 10.1001/jama.288.6.728

[107] Johnson S, Hennessy E, Smith R, Trikic R, Wolke D, Marlow N. Academic attainment and special educational needs in extremely preterm children at 11 years of age: the EPICure study. Archives of Disease in Childhood—Fetal and Neonatal Edition. 2009;94(4):F283–F9. 10.1136/adc.2008.152793 19282336

[108] Kovachy VN, Adams JN, Tamaresis JS, Feldman HM. Reading abilities in school-aged preterm children: a review and meta-analysis. Dev Med Child Neurol. 2014 10.1111/dmcn.12652 . 25516105 PMC4397135

[109] Luu TM, Ment LR, Schneider KC, Katz KH, Allan WC, Vohr BR. Lasting effects of preterm birth and neonatal brain hemorrhage at 12 years of age. Pediatrics. 2009;123(3):1037–44. 10.1542/peds.2008-1162 19255037 PMC2651566

[110] Yakovlev P, Lecours A. The myelogenetic cycles of regional maturation of the brain Minkowski A, editor: Oxford, Blackwell; 1967.

[111] Giedd JN, Blumenthal J, Jeffries NO, Castellanos FX, Liu H, Zijdenbos A, et al Brain development during childhood and adolescence: a longitudinal MRI study. Nat Neurosci. 1999;2(10):861–3. 10491603 10.1038/13158

[112] Allen MC. Neurodevelopmental outcomes of preterm infants. Curr Opin Neurol. 2008;21(2):123–8. 10.1097/WCO.0b013e3282f88bb4 18317268

[113] Kidokoro H, Anderson PJ, Doyle LW, Woodward LJ, Neil JJ, Inder TE. Brain injury and altered brain growth in preterm infants: predictors and prognosis. Pediatrics. 2014;134(2):e444–e53. 10.1542/peds.2013-2336 25070300

[114] Groppo M, Ricci D, Bassi L, Merchant N, Doria V, Arichi T, et al Development of the optic radiations and visual function after premature birth. Cortex. 2014;56:30–7. Epub 2012/04/10. 10.1016/j.cortex.2012.02.008S0010-9452(12)00064-0 [pii]. . 22482694

[115] Van Pul C, Van Kooij B, De Vries L, Benders M, Vilanova A, Groenendaal F. Quantitative FIber Tracking in the Corpus Callosum and internal capsule reveals microstructural abnormalities in preterm infants at term equivalent age. AJNR. 2012;33:678–784. 10.3174/ajnr.A2859 22194382 PMC8050470

[116] Ball G, Srinivasan L, Aljabar P, Counsell SJ, Durighel G, Hajnal JV, et al Development of cortical microstructure in the preterm human brain. Proc Natl Acad Sci U S A. 2013;110(23):9541–6. 10.1073/pnas.1301652110 23696665 PMC3677430

[117] Pandit AS, Robinson E, Aljabar P, Ball G, Gousias IS, Wang Z, et al Whole-brain mapping of structural connectivity in infants reveals altered connection strength associated with growth and preterm birth. Cereb Cortex. 2014;24(9):2324–33. 10.1093/cercor/bht086 . 23547135

[118] Ball G, Boardman JP, Rueckert D, Aljabar P, Arichi T, Merchant N, et al The effect of preterm birth on thalamic and cortical development. Cereb Cortex. 2012;22(5):1016–24. 10.1093/cercor/bhr176 21772018 PMC3328341

[119] Ball G, Counsell SJ, Anjari M, Merchant N, Arichi T, Doria V, et al An optimised tract-based spatial statistics protocol for neonates: applications to prematurity and chronic lung disease. Neuroimage. 2010;53(1):94–102. 10.1016/j.neuroimage.2010.05.055 . 20510375

[120] Tyson JE, Parikh NA, Langer J, Green C, Higgins RD, National Institute of Child H, et al Intensive care for extreme prematurity—moving beyond gestational age. N Engl J Med. 2008;358(16):1672–81. 10.1056/NEJMoa073059 18420500 PMC2597069

[121] Ambalavanan N, Carlo WA, Tyson JE, Langer JC, Walsh MC, Parikh NA, et al Outcome trajectories in extremely preterm infants. Pediatrics. 2012;130(1):e115–25. 10.1542/peds.2011-3693 22689874 PMC3382921

[122] Vohr BR. Neurodevelopmental outcomes of extremely preterm infants. Clin Perinatol. 2014;41(1):241–55. 10.1016/j.clp.2013.09.003 . 24524458

[123] Wedeen VJ, Wang RP, Schmahmann JD, Benner T, Tseng WY, Dai G, et al Diffusion spectrum magnetic resonance imaging (DSI) tractography of crossing fibers. Neuroimage. 2008;41(4):1267–77. 10.1016/j.neuroimage.2008.03.036 . 18495497

[124] Tuch DS, Reese TG, Wiegell MR, Makris N, Belliveau JW, Wedeen VJ. High angular resolution diffusion imaging reveals intravoxel white matter fiber heterogeneity. Magn Reson Med. 2002;48(4):577–82. 10.1002/mrm.10268 . 12353272

[125] Deoni SC, Rutt BK, Arun T, Pierpaoli C, Jones DK. Gleaning multicomponent T1 and T2 information from steady-state imaging data. Magn Reson Med. 2008;60(6):1372–87. 10.1002/mrm.21704 . 19025904

[126] Mezer A, Yeatman JD, Stikov N, Kay KN, Cho NJ, Dougherty RF, et al Quantifying the local tissue volume and composition in individual brains with magnetic resonance imaging. Nat Med. 2013;19(12):1667–72. Epub 2013/11/05. 10.1038/nm.3390 nm.3390 [pii]. 24185694 PMC3855886

[127] Qiu A, Rifkin-Graboi A, Zhong J, Phua DY, Lai YK, Meaney MJ. Birth weight and gestation influence striatal morphology and motor response in normal six-year-old boys. Neuroimage. 2012;59(2):1065–70. 10.1016/j.neuroimage.2011.09.032 . 21963914

[128] Walhovd KB, Fjell AM, Brown TT, Kuperman JM, Chung Y, Hagler DJ Jr, et al Long-term influence of normal variation in neonatal characteristics on human brain development. Proc Natl Acad Sci U S A. 2012;109(49):20089–94. 10.1073/pnas.1208180109 23169628 PMC3523836

