# Supplementary material for: Decreased and Increased Anisotropy along Major Cerebral White Matter Tracts in Preterm Children and Adolescents
Source: PLoS One. 2015 Nov 11;10(11):e0142860. doi: 10.1371/journal.pone.0142860 (PMC4641645; doi:10.1371/journal.pone.0142860)
Supplement: S1 Fig — (DOCX) [file pone.0142860.s001.docx]

**S1 Fig. Spearman correlation between GA and mean FA from the right IFOF where significant group differences were observed (location 18-19).**

GA = gestational age; BW = birth weight; IFOF = inferior frontal occipital fasciculus; R= right; rs = Spearman correlation coefficient; * p < 0.05.
