# Supplementary material for: Decreased and Increased Anisotropy along Major Cerebral White Matter Tracts in Preterm Children and Adolescents
Source: PLoS One. 2015 Nov 11;10(11):e0142860. doi: 10.1371/journal.pone.0142860 (PMC4641645; doi:10.1371/journal.pone.0142860)
Supplement: S1 Table — (DOCX) [file pone.0142860.s002.docx]

**Fiber Tracking**

The number of full term and preterm subjects in which fiber reconstruction was performed successfully is reported for each tract in S1 Table.

**S1 Table**. **Fiber Tract Identification in Full Term and Preterm Subjects**

| Tract Name | Preterm  (*n*=27) | | Full Term  (*n*=19) | |  |
| --- | --- | --- | --- | --- | --- |
|  | *n* | *n* | % | % | |
| Arc-L | 26 | 19 | 100 | 96.3 | |
| Arc-R | 23 | 13 | 68.4 | 85.2 | |
| CST-L | 27 | 19 | 100 | 100 | |
| CST-R | 26 | 19 | 100 | 96.3 | |
| FMajor | 26 | 19 | 100 | 96.3 | |
| FMinor | 27 | 19 | 100 | 100 | |
| UF-L | 27 | 19 | 100 | 100 | |
| UF-R | 27 | 19 | 100 | 100 | |
| ATR-L | 27 | 19 | 100 | 100 | |
| ATR-R | 27 | 19 | 100 | 100 | |
| Cing-L | 26 | 19 | 100 | 96.3 | |
| Cing-R | 26 | 19 | 100 | 96.3 | |
| IFOF-L | 27 | 19 | 100 | 100 | |
| IFOF-R | 27 | 19 | 100 | 100 | |
| ILF-L | 27 | 19 | 100 | 100 | |
| ILF-R | 27 | 19 | 100 | 100 | |
| aSLF-L | 27 | 19 | 100 | 100 | |
| aSLF-R | 27 | 19 | 100 | 100 | |

Arc = Arcuate Fasciculus; CST = Corticospinal Tract; FMajor = Forceps Major; FMinor = Forceps Minor; UF = Uncinate Fasciculus; ATR = Anterior Thalamic Radiation; Cing = Cingulum; IFOF = Inferior Fronto-occipital Fasciculus; ILF = Inferior Longitudinal Fasciculus; aSLF = Anterior Superior Longitudinal Fasciculus
