# Supplementary material for: Decreased and Increased Anisotropy along Major Cerebral White Matter Tracts in Preterm Children and Adolescents
Source: PLoS One. 2015 Nov 11;10(11):e0142860. doi: 10.1371/journal.pone.0142860 (PMC4641645; doi:10.1371/journal.pone.0142860)
Supplement: S2 Table — (DOCX) [file pone.0142860.s003.docx]

**S2 Table. Spearman Correlations between mean FA of 18 Cerebral White Matter Tracts and Age at Diffusion Imaging for Preterm and Full Term Groups**.

| Tract | Preterm | | | Full Term | |
| --- | --- | --- | --- | --- | --- |
|  | *rs* | *p* | *rs* | | *p* |
| Arc |  |  |  | |  |
| Left | 0.15, | 0.46 | 0.16, | | 0.36 |
| Right | -0.05, | 0.82 | 0.63, | | 0.02* |
| CST |  |  |  | |  |
| Left | 0.08, | 0.70 | 0.33, | | 0.17 |
| Right | -0.13, | 0.53 | 0.15, | | 0.53 |
| FMajor |  |  |  | |  |
| Left | 0.02, | 0.92 | -0.01, | | 0.97 |
| Right | 0.12, | 0.55 | -0.07, | | 0.78 |
| FMinor |  |  |  | |  |
| Left | -0.05, | 0.81 | 0.04, | | 0.86 |
| Right | 0.15, | 0.45 | -0.10, | | 0.68 |
| UF |  |  |  | |  |
| Left | -0.22, | 0.26 | 0.22, | | 0.36 |
| Right | -0.16, | 0.44 | 0.23, | | 0.36 |
| ATR |  |  |  | |  |
| Left | 0.07, | 0.73 | 0.27, | | 0.27 |
| Right | 0.09, | 0.66 | 0.07, | | 0.78 |
| Cing |  |  |  | |  |
| Left | -.013, | 0.54 | 0.44, | | 0.06+ |
| Right | 0.10, | 0.62 | 0.45, | | 0.05+ |
| IFOF |  |  |  | |  |
| Left | -0.17, | 0.40 | 0.16, | | 0.51 |
| Right | -0.02, | 0.92 | 0.26, | | 0.28 |
| ILF |  |  |  | |  |
| Left | 0.36, | 0.06+ | 0.11, | | 0.65 |
| Right | 0.03, | 0.87 | -0.00, | | 0.99 |
| aSLF |  |  |  | |  |
| Left | -0.02, | 0.93 | 0.05, | | 0.84 |
| Right | 0.30, | 0.13 | 0.11, | | 0.67 |

Arc = Arcuate Fasciculus; CST = Corticospinal Tract; FMajor = Forceps Major; FMinor = Forceps Minor; UF = Uncinate Fasciculus; ATR = Anterior Thalamic Radiation; Cing = Cingulum; IFOF = Inferior Fronto-occipital Fasciculus; ILF = Inferior Longitudinal Fasciculus; aSLF = Anterior Superior Longitudinal Fasciculus

**p* < 0.05, + trend *p* < 0.1
