# Supplementary material for: Decreased and Increased Anisotropy along Major Cerebral White Matter Tracts in Preterm Children and Adolescents
Source: PLoS One. 2015 Nov 11;10(11):e0142860. doi: 10.1371/journal.pone.0142860 (PMC4641645; doi:10.1371/journal.pone.0142860)
Supplement: S3 Table — (DOCX) [file pone.0142860.s004.docx]

**Table S3. Spearman Correlations between mean MD of 18 Cerebral White Matter Tracts and Age at Diffusion Imaging for Preterm and Full Term Groups.**

| Tract | Preterm | | | Full Term | |
| --- | --- | --- | --- | --- | --- |
|  | *rs* | *p* | *rs* | | *p* |
| Arc |  |  |  | |  |
| Left | 0.09, | 0.66 | -0.61, | | 0.006* |
| Right | 0.03, | 0.88 | -0.53, | | 0.06+ |
| CST |  |  |  | |  |
| Left | 0.02, | 0.94 | -0.32, | | 0.18 |
| Right | 0.10, | 0.63 | -0.29, | | 0.23 |
| FMajor |  |  |  | |  |
| Left | 0.00, | 0.99 | 0.05, | | 0.85 |
| Right | -0.24, | 0.24 | 0.03, | | 0.92 |
| FMinor |  |  |  | |  |
| Left | 0.08, | 0.70 | -0.32, | | 0.19 |
| Right | -0.19, | 0.36 | -0.24, | | 0.32 |
| UF |  |  |  | |  |
| Left | -0.09, | 0.65 | -0.51, | | 0.03* |
| Right | 0.04, | 0.83 | -0.57, | | 0.01* |
| ATR |  |  |  | |  |
| Left | 0.13, | 0.53 | -0.27, | | 0.27 |
| Right | 0.10, | 0.62 | -0.00, | | 0.99 |
| Cing |  |  |  | |  |
| Left | 0.13, | 0.54 | -0.63, | | 0.004* |
| Right | -0.11, | 0.61 | -0.47, | | 0.05+ |
| IFOF |  |  |  | |  |
| Left | 0.29, | 0.14 | -0.50, | | 0.03* |
| Right | 0.42, | 0.03* | -0.54, | | 0.02 |
| ILF |  |  |  | |  |
| Left | -0.13, | 0.52 | -0.54, | | 0.02* |
| Right | 0.18, | 0.37 | -0.67, | | 0.002* |
| aSLF |  |  |  | |  |
| Left | -0.04, | 0.86 | -0.50, | | 0.03* |
| Right | -0.06, | 0.78 | -0.57, | | 0.01* |

**p* < 0.05, + trend *p* < 0.1

Arc = Arcuate Fasciculus; CST = Corticospinal Tract; FMajor = Forceps Major; FMinor = Forceps Minor; UF = Uncinate Fasciculus; ATR = Anterior Thalamic Radiation; Cing = Cingulum; IFOF = Inferior Fronto-occipital Fasciculus; ILF = Inferior Longitudinal Fasciculus; aSLF = Anterior Superior Longitudinal Fasciculus
