# Supplementary material for: Decreased and Increased Anisotropy along Major Cerebral White Matter Tracts in Preterm Children and Adolescents
Source: PLoS One. 2015 Nov 11;10(11):e0142860. doi: 10.1371/journal.pone.0142860 (PMC4641645; doi:10.1371/journal.pone.0142860)
Supplement: S4 Table — (DOCX) [file pone.0142860.s005.docx]

**S4 Table. Group comparison between mean tract volumes of 18 cerebral white matter tracts and whole brain fiber group.**

| Tract | Preterm Mean  Volume (std)  mm^3^ | | Full Term Mean  Volume (std)  mm^3^ | | t-stat | *p-val* |
| --- | --- | --- | --- | --- | --- | --- |
| Arc-L | 1965.31 | (937.53) | 2057.32 | (909.41) | 0.33 | 0.74 |
| Arc-R | 1082.57 | (623.25) | 1431.43 | (746.76) | 1.47 | 0.16 |
| CST-L | 5504.78 | (1498.99) | 6177.37 | (1138.55) | 1.73 | 0.09 |
| CST-R | 5924.35 | (965.32) | 6174.26 | (1448.70) | 0.65 | 0.52 |
| FMajor | 5277.77 | (2516.53) | 6985.21 | (1740.39) | 2.69 | 0.01 |
| FMinor | 10828.04 | (2618.82) | 11652.79 | (1791.21) | 1.27 | 0.21 |
| UF-L^++^ | 1628.37 | (501.60) | 1656.26 | (346.08) | 0.223 | 0.82 |
| UF-R^++^ | 1647.78 | (532.86) | 1750.16 | (570.79) | 0.62 | 0.54 |
| ATR-L^+^ | 5311.78 | (1364.38) | 5638.32 | (928.06) | 0.97 | 0.34 |
| ATR-R^+^ | 5367.56 | (1354.25) | 5144.68 | (991.70) | -0.73 | 0.47 |
| Cing-L | 1586.56 | (579.95) | 1594.42 | (379.83) | 0.06 | 0.96 |
| Cing-R | 1618.69 | (709.48) | 1354.68 | (411.38) | -1.57 | 0.12 |
| IFOF-L | 4739.0 | (1815.80) | 4448.79 | (1426.21) | -0.61 | 0.55 |
| IFOF-R^+/++^ | 3942.63 | (1078.66) | 4591.16 | (850.37) | 2.28 | 0.03 |
| ILF-L^+^ | 4795.33 | (1306.61) | 4819.53 | (1605.16) | 0.05 | 0.96 |
| ILF-R^+^ | 4676.48 | (1492.00) | 4004.26 | (1292.66) | -1.63 | 0.11 |
| aSLF-L | 2687.04 | (1111.99) | 2321.95 | (856.17) | -1.26 | 0.22 |
| aSLF-R | 3575.26 | (1158.32) | 3105.12 | (1020.41) | -1.45 | 0.13 |
| Whole-Brain | 698227.21 | (64910.94) | 713104.11 | (75136.89) | -0.72 | 0.48 |

+ indicates tracts where FA was significantly higher in the PT as compared to the FT group;

++ indicates tracts where FA was significantly lower in the PT as compared to the FT group

Arc = Arcuate Fasciculus; CST = Corticospinal Tract; FMajor = Forceps Major; FMinor = Forceps Minor; UF = Uncinate Fasciculus; ATR = Anterior Thalamic Radiation; Cing = Cingulum; IFOF = Inferior Fronto-occipital Fasciculus; ILF = Inferior Longitudinal Fasciculus; aSLF = Anterior Superior Longitudinal Fasciculus; L = Left; Right = Right
