# Supplementary material for: Decreased and Increased Anisotropy along Major Cerebral White Matter Tracts in Preterm Children and Adolescents
Source: PLoS One. 2015 Nov 11;10(11):e0142860. doi: 10.1371/journal.pone.0142860 (PMC4641645; doi:10.1371/journal.pone.0142860)
Supplement: S5 Table — (DOCX) [file pone.0142860.s006.docx]

**S5 Table. ANCOVA results for the comparison of preterm and full term MD profiles**

| Tract | Main Effect of Group | Group by Location Interaction | Group by Hemisphere Interaction |
| --- | --- | --- | --- |
| Arc | F = 3.26, p = 0.08 | F = 1.87, p = 0.09 | F = 0.49, p = 0.49 |
| CST | F = 0.04, p = 0.83 | F = 2.02, p = 0.11 | F = 0.86, p = 0.36 |
| FMajor | F = 1.91, p = 0.17 | F = 4.29, p < 0.01** | F = 0.01, p = 0.92 |
| FMinor | F = 0.02, p = 0.90 | F = 4.24, p = 0.01** | F = 2.00, p = 0.17 |
| UF | F = 1.18, p = 0.28 | F = 1.20, p = 0.31 | F = 0.10, p = 0.76 |
| ATR | F = 1.10, p = 0.30 | F = 0.68, p = 0.51 | F = 2.60, p = 0.11 |
| Cing | F = 0.96, p = 0.33 | F = 1.19, p = 0.32 | F = 2.92, p = 0.09 |
| IFOF | F = 0.20, p = 0.67 | F = 0.84, p = 0.43 | F = 3.36, p = 0.07 |
| ILF | F = 4.51, p = 0.04* | F = 2.02, p = 0.18 | F = 2.85, p = 0.10 |
| aSLF | F = 0.96, p = 0.33 | F = 0.36, p = 0.73 | F = 2.62, p = 0.11 |

**p* < 0.05, ***p* < 0.01, uncorrected

Arc = Arcuate Fasciculus; CST = Corticospinal Tract; FMajor = Forceps Major; FMinor = Forceps Minor; UF = Uncinate Fasciculus; ATR = Anterior Thalamic Radiation; Cing = Cingulum; IFOF = Inferior Fronto-occipital Fasciculus; ILF = Inferior Longitudinal Fasciculus; aSLF = Anterior Superior Longitudinal Fasciculus
