# Supplementary material for: Decreased and Increased Anisotropy along Major Cerebral White Matter Tracts in Preterm Children and Adolescents
Source: PLoS One. 2015 Nov 11;10(11):e0142860. doi: 10.1371/journal.pone.0142860 (PMC4641645; doi:10.1371/journal.pone.0142860)
Supplement: S1 Text — (DOCX) [file pone.0142860.s007.docx]

**Supporting Information**

**Translation Head Motion**

Group comparisons were performed in order to ensure that preterm and full term groups did not differ in terms of the amount of translational head motion detected during image preprocessing of diffusion images. This was achieved by computing, for each subject, the magnitude (in millimeters) of motion correction required for each image volume and each image plane (x-y-z). From these data, we generated a mean motion value for each image plane from all individuals of the control group and from all individuals of the preterm group. We computed unpaired t-tests to examine whether the preterm and full term groups differed on the basis of translational head motion in each plane. Overall, we found that the average translational head motion was minimal in both groups (<1.5mm) and was not significantly different between groups for any image plane p > 0.05.
